# New insights into polyploid evolution and dynamic nature of *Ludwigia* section *Isnardia* (Onagraceae)

**DOI:** 10.1186/s40529-023-00387-8

**Published:** 2023-06-03

**Authors:** Shih-Hui Liu, Kuo-Hsiang Hung, Tsai-Wen Hsu, Peter C. Hoch, Ching-I Peng, Tzen-Yuh Chiang

**Affiliations:** 1 Department of Biological Sciences, National Sun Yat-Sen University, Kaohsiung, 804 Taiwan; 2 Graduate Institute of Bioresources, National Pingtung University of Science and Technology, Pingtung, 912 Taiwan; 3 Endemic Species Research Institute, Nantou, 552 Taiwan; 4 Missouri Botanical Garden, St. Louis, MO 63166 USA; 5 Biodiversity Research Center, Academia Sinica, Taipei, 11529 Taiwan; 6 Department of Life Sciences, National Cheng Kung University, Tainan, 701 Taiwan

**Keywords:** Divergence time, Genetic variation, Hybridization, Interspecific gene flow, Polyploid evolution

## Abstract

**Background:**

While polyploids are common in plants, the evolutionary history and natural dynamics of most polyploid groups are still unclear. Owing to plentiful earlier systematic studies, *Ludwigia* sect. *Isnardia* (comprising 22 wetland taxa) is an ideal allopolyploid complex to investigate polyploid evolution and natural dynamics within and among taxa. With a considerable sampling, we concentrated on revisiting earlier phylogenies of *Isnardia*, reevaluating the earlier estimated age of the most recent common ancestor (TMRCA), exploring the correlation between infraspecific genetic diversity and ploidy levels, and inspecting interspecific gene flows among taxa.

**Results:**

Phylogenetic trees and network concurred with earlier phylogenies and hypothesized genomes by incorporating 192 atpB-rbcL and ITS sequences representing 91% of Isnardia taxa. Moreover, we detected three multi-origin taxa. Our findings on *L. repens* and *L. sphaerocarpa* were consistent with earlier studies; *L. arcuata* was reported as a multi-origin taxon here, and an additional evolutionary scenario of *L. sphaerocarpa* was uncovered, both for the first time. Furthermore, estimated *Isnardia* TMRCA ages based on our data (5.9 or 8.9 million years ago) are in accordance with earlier estimates, although younger than fossil dates (Middle Miocene). Surprisingly, infraspecific genetic variations of *Isnardia* taxa did not increase with ploidy levels as anticipated from many other polyploid groups. In addition, the exuberant, low, and asymmetrical gene flows among *Isnardia* taxa indicated that the reproductive barriers may be weakened owing to allopolyploidization, which has rarely been reported.

**Conclusions:**

The present research gives new perceptions of the reticulate evolution and dynamic nature of *Isnardia* and points to gaps in current knowledge about allopolyploid evolution.

**Supplementary Information:**

The online version contains supplementary material available at 10.1186/s40529-023-00387-8.

## Background

Polyploidization has long been recognized as an essential force in plant evolution (Raven and Thompson 1964; Leitch and Bennett 1997; Soltis et al. 2014); however, the complex nature of polyploids has restrained research efforts on these plants (Soltis et al. 2009; Dufresne et al. 2014; Baduel et al. 2018). Many questions about polyploid evolution are not yet thoroughly resolved. For example, how did polyploidization occur? How many times has polyploidization occurred in the history of a specific group? Do polyploids interact with closely related taxa; if yes, how? Is a polyploid taxon diverse at the molecular level; if yes, how? An increasing number of studies are attempting to dissect the origins of polyploids and the dynamic nature among and within polyploid taxa by applying the rapidly developing molecular and computational techniques (e.g. Van de Peer et al. 2021; Karbstein et al. 2022; Zhuang et al. 2022). *Ludwigia* L. section *Isnardia* (L.) W.L. Wagner & Hoch, a polyploid complex with a moderate number of species and sufficient knowledge from plenty of earlier systematic studies, is an ideal plant group to explore polyploid phylogeny as well as the dynamic nature of polyploids.

*Isnardia* currently contains 22 taxa, including five diploids, 11 tetraploids, four hexaploids, one octoploid, and one taxon with an unknown ploidy level (Table 1) (Raven 1963; Raven and Tai 1979; Peng 1988, 1989; Peng et al. 2005; Wagner et al. 2007; Arya et al. 2020; Liu et al. 2020). *Isnardia* are wetland weeds characterized by their creeping or erect stem, 4-merous and haplostemonous flowers, terete or globose capsules, and pluriseriate and free seeds (Wagner et al. 2007). Members of *Isnardia* were previously classified into three sects. *Isnardia*, *Michelia* Ramamoorthy, and *Microcarpium* Munz (Munz 1944; Raven 1963; Ramamoorthy 1979; Wagner et al. 2007). Early systematic studies have suggested that these three sects. are closely related and, together, compose the *Microcarpium* complex (Eyde 1977, 1978, 1981; Peng and Tobe 1987; Peng et al. 1988, 2005; Tobe et al. 1988; Peng 1989; Wagner et al. 2007). Based on morphological and anatomic studies, this complex was proposed to be evolutionarily closed to sect. *Ludwigia* L. (Eyde 1977, 1978, 1981). Recent molecular phylogenies revealed that this complex is a monophyletic group with strong (Liu et al. 2020) or weak (Liu et al. 2017) support, and sect. *Ludwigia* is sister to the complex (Liu et al. 2017, 2020). However, none of the three sects. in the complex exhibit monophyly (Hung et al. 2009; Liu et al. 2017, 2020), therefore the three sects. in the complex were combined into the sect. *Isnardia* (Liu et al. 2020). Examining the combination with different data sets will provide valuable information on this taxonomic treatment.

**Table 1:** Sampled *Ludwigia* sect. *Isnardia* taxa, their sample sizes, genome types, ploidy levels, and nucleotide diversities (Tajima’s π) of the studied regions

Taxon	Sample size (ITS/* atp*B-*rbc*L)	Genome type	Ploidy level	Nucleotide diversity (π)	
				ITS	*atp*B-*rbc*L
*Ludwigia alata* Elliott	9/8	AABBDD	6 ×	0.00371 ± 0.00140	0.00173 ± 0.00073
*Ludwigia arcuata* Walter	4/3	BBCC	4 ×	0.00809 ± 0.00261	0.00164 ± 0.00117
*Ludwigia brevipes* (Long) Eame	6/1	AABBCC	6 ×	0.00370 ± 0.00176	–
*Ludwigia curtissii* Chapm	5/5	BBCCDDF_1_F_1_	8 ×	0.00063 ± 0.00061	0.00098 ± 0.00068
*Ludwigia glandulosa* subsp*. brachycarpa* (Torr. & A. Gray) C.I. Peng	3/3	AABB	4 ×	0.00105 ± 0.00106	0.00082 ± 0.00083
*Ludwigia glandulosa* Walter subsp. *glandulosa*	5/4	AABB	4 ×	0.00190 ± 0.00113	0.00246 ± 0.00146
*Ludwigia lanceolata* Elliott	3/3	AABB	4 ×	0.01082 ± 0.00356	0.00702 ± 0.00242
*Ludwigia linearis* Walter	4/4	F_2_F_2_	2 ×	0.01379 ± 0.00354	0.00081 ± 0.00080
*Ludwigia linifolia* Poir	5/4	F_1_F_1_	2 ×	0.00517 ± 0.00187	0.00000 ± 0.00000
*Ludwigia microcarpa* Michx	6/6	DD	2 ×	0.00000 ± 0.00000	0.00000 ± 0.00000
*Ludwigia ovalis* Miq	5/7	M_1_M_1_M_2_M_2_	4 ×	0.00094 ± 0.00093	0.00432 ± 0.00141
*Ludwigia palustris* (L.) Elliott	6/3	AA	2 ×	0.00189 ± 0.00115	0.00246 ± 0.00141
*Ludwigia pilosa* Walter	7/5	AABB	4 ×	0.00444 ± 0.00157	0.01896 ± 0.00330
*Ludwigia polycarpa* Short & R.Peter	4/4	AABB	4 ×	0.00559 ± 0.00220	0.00247 ± 0.00125
*Ludwigia ravenii* C.I Peng	2/4	AABB	4 ×	0.00798 ± 0.00364	0.00041 ± 0.00040
*Ludwigia repens* J.R. Forst	9/3	AABBCC	6 ×	0.00667 ± 0.00180	0.00246 ± 0.00141
*Ludwigia simpsonii* Chapm	3/3	BBCCDD	6 ×	0.02097 ± 0.00476	0.00082 ± 0.00086
*Ludwigia spathulata* Torr. & A. Gray	2/3	AADD	4 ×	0.00000 ± 0.00000	0.00163 ± 0.00110
*Ludwigia sphaerocarpa* Elliott	5/5	AABB	4 ×	0.00939 ± 0.00261	0.00148 ± 0.00084
*Ludwigia suffruticosa* Walter	5/4	AABB	4 ×	0.01121 ± 0.00288	0.00413 ± 0.00158
Sect. *Isnardia* (L.) W. L. Wagner & Hoch	98/82			0.00590 ± 0.00195	0.00273 ± 0.00108

The ploidy levels and genome types were adopted from earlier cytological and molecular studies (Raven and Tai 1979; Peng 1988, 1989; Peng et al. 2005; Liu et al. 2020). The em dashes indicate unavailable data because only one sample was applied for the taxon

Extant *Isnardia* plants are mainly distributed in North America with two exceptions––*L. ovalis* Miq. is endemic to East Asia and *L. venugopalanii* S. Arya, V. Suresh, P. Biju & V.S.A. Kumar occurs in India––and one widely spread taxon, *L. palustris* (L.) Elliott, which occurrs in North, Central, and South America, Eurasia, and Africa (Raven 1963; Peng et al. 2005; Wagner et al. 2007; Arya et al. 2020). Geographical distribution and phylogeographic analyses indicated that *Isnardia* plants originated in North America and a few taxa spread to other continents later (Raven 1963; Eyde 1981; Tobe et al. 1988; Liu et al. 2020). The earliest known fossil record of *Isnardia* indicated that this clade had extended to Europe in Middle Miocene (Friis 1985; Tobe et al. 1988) (ca. 11.63–15.97 million years ago (MYA) referring to the International Commission on Stratigraphy (ICS), https://stratigraphy.org/). That is, *Isnardia* likely originated before Middle Miocene. However, the results of Hung et al. (2009)’s coalescence analyses suggested a younger the most recent common ancestor (TMRCA) of *Isnardia*, which was 5.99 ± 0.02 MYA based on chloroplast *atp*B-*rbc*L data and 6.59 ± 0.02 MYA based on nuclear ITS data. Additional molecular data, more thorough sampling, and further analyses are warranted to give a better perception of the divergence time of *Isnardia*.

Origins of *Isnardia* taxa have been intensively investigated using morphological, anatomic, cytological, and molecular data (Raven 1963; Eyde 1977, 1978, 1981; Raven and Tai 1979; Peng and Tobe 1987; Tobe et al. 1988; Peng 1988, 1989; Peng et al. 2005; Hung et al. 2009; Liu et al. 2017, 2020). Eight ancestral genomes––including genomes A, B (= H), C (= I), D (= G), F_1_, F_2_, M_1_, and M_2_––have been proposed and assigned to each *Isnardia* taxon (Table 1) based on cytological and molecular works (Peng 1988, 1989; Peng et al. 2005; Hung et al. 2009; Liu et al. 2020). The reticulate origins of the 16 allopolyploid *Isnardia––*consisting of 11 tetraploids, four hexaploids, and one octoploid (Table 1)––were also inferred (Liu et al. 2020). Multiple origins are common in polyploid speciation (Soltis and Soltis 1993; Leitch and Bennett 1997; Peng and Chiang 2000; Doyle et al. 2004). To date, however, only two *Isnardia*––tetraploid *L. sphaerocarpa* Elliott and hexaploid *L. repens* J.R. Forst.––have been considered to have multiple origins (Peng 1988; Liu et al. 2020), because of the limitation of small sample sizes for each taxon in the earlier studies. Comprehensively decoding the polyploid *Isnardia* taxa's origins requires a larger sample size for each taxon*.*

In addition, higher genetic diversities have been detected in the taxa with higher ploidy levels in several plant groups (e.g. Mallet 2007; García‐Verdugo et al. 2009; Bogačiovienė et al. 2019; Zhang et al. 2019). At the same time, earlier studies have observed plenty of natural hybrids among *Isnardia* taxa in the field and reported the high capability of interspecific hybridization among *Isnardia* taxa in breeding experiments at research greenhouses (Raven and Tai 1979; Peng 1988, 1989; Peng et al. 2005). However, these attributes of *Isnardia* taxa have not been well explored. Hung et al. (2009) measured infraspecific genetic diversities of six *Isnardia* taxa. Both Liu et al. (2017) and Liu et al. (2020) sampled 20 *Isnardia* taxa, but they could not approximate interspecific gene flow nor infraspecific genetic diversities due to small sample sizes for each taxon. Moreover, a larger sample size for each taxon will provide a better insight into these characteristics of *Isnardia*.

In the present study, we intended to meet the following four aims by comprehensively sampling *Isnardia*. First, we reexamined the reticulate evolution hypotheses implied by earlier studies (Peng 1988, 1989; Peng et al. 2005; Hung et al. 2009; Liu et al. 2020) and investigated multiple origins of polyploid taxa. Second, we reevaluated the estimated ages of *Isnardia* TMRCA (Hung et al. 2009). Third, we tested the proposition that, as with many other allopolyploid plant groups (Mallet 2007; García‐Verdugo et al. 2009; Bogačiovienė et al. 2019; Zhang et al. 2019), genetic diversities of *Isnardia* taxa increase with their ploidy levels. Fourth, consistent with earlier field observations and breeding experiments (Raven and Tai 1979; Peng 1988, 1989; Peng et al. 2005), we investigated the interspecific gene flows among *Isnardia* taxa with different ploidy levels.

## Methods

### Sampling

We aimed to sample all *Isnardia* taxa. For each *Isnardia* taxon, we planned to sample three to ten individuals. Living plants were collected during collecting trips in Alabama, Florida, Massachusetts, Missouri, North Carolina, Oklahoma, South Carolina, Tennessee, Texas, and Virginia in the USA. Leaf tissue for genomic DNA extraction was dried in silica gel immediately after being collected. Vouchers were deposited at the Herbarium of Endemic Species Research Institute (TAIE) (Thiers 2016), Taiwan, for further studies. Samples of the Cuban endemic species, *L. stricta* (C. Wright ex Griseb.) C. Wright, were obtained from the herbarium vouchers provided by the Herbarium of Missouri Botanical Garden (MO) (Thiers 2016), Saint Louis, Missouri, USA. Unfortunately, samples for the recently described Indian endemic species, *L. venugopalanii* (Arya et al. 2020), were unavailable. Additional *Isnardia* taxa from Hung et al. (2009) were also incorporated into this study. In addition, published DNA sequences of some outgroups from other sections in *Ludwigia* and other genera in Onagraceae were downloaded from the GenBank (Sayers et al. 2019) for the analyses.

### Genomic DNA extraction, PCR, cloning, sequencing, and assembly

The genomic DNA of our samples was extracted by optimizing the cetyltrimethylammonium bromide (CTAB) method (Murray and Thompson 1980; Doyle and Doyle 1987) for *Isnardia* samples. A mixture of 5–10 mg ground leaf tissue, 10 mL 65 °C 3X CTAB isolation buffer, and 40 μL 0.4% ß-mercaptoethanol was incubated at 65 °C for 30 min. After the incubation, 10 mL chloroform-isoamyl alcohol (24:1) was added to the mixture. The solution was gently, thoroughly mixed, and then centrifuged at 6000×*g* for 10 min at about 20 °C. The supernatant was retained, and the process described in the previous sentence was repeated twice. The final supernatant was mixed well with 10 mL isopropanol, incubated at − 20 °C for 30 min to 24 h, warmed up, and centrifuged at 15,000×*g* for 10 min at 4 °C. The precipitated DNA was air-dried, dissolved with 500 μL TE buffer, and incubated at 37 °C for 30 min with RNase A. A 500 μL isopropanol was applied to precipitate DNA again. The precipitated DNA was then resuspended with 70% ethanol, centrifuged at 15,000×*g* for 2–3 min at 4 °C, air-dried, and dissolved in 200 μL TE buffer for the following polymerase chain reactions (PCR).

Both nuclear ITS (ITS1 + 5.8S + ITS2) and chloroplast *atp*B-*rbc*L regions were amplified with the universal primers (White et al. 1990; Chiang et al. 1998; Hung et al. 2009) for all *Isnardia* samples. A 100 μL mixture including 0.5 μL 10U/*μL* Taq Polymerase (Promega, Madison, Wisconsin, USA), 10 μL 10X PCR buffer, 10 μL 8 mM dNTP, 10 μL 2 pM forward primer, 10 μL 2 pM reverse primer, 10 μL 10 mM MgCl_2_, 10 μL 2 ng/ μL genomic DNA, and distilled water was applied in each PCR amplification. The genomic DNA in the mixture was initially denatured at 92 °C for 5 min, followed by 31 cycles of 92 °C for 45 s, 53 °C for 75 s, and 72 °C for 90 s, and finally elongated at 72 °C for 10 min. The PCR products were then purified with 1% agarose gel and the Gel/PCR DNA Isolation System (Viogene, Taipei, Taiwan).

In the case where multiple sizes of PCR products were amplified for one individual, TA cloning was applied using the pGEM-T Easy Cloning Vector (Promega, Madison, Wisconsin, USA) to determine variations within the individual.

Subsequently, PCR products and colonies were sequenced on an ABI 3730XL DNA Analyzer (Applied Biosystems, Waltham, Massachusetts, USA) commercially with the universal primers (White et al. 1990; Chiang et al. 1998; Hung et al. 2009). DNA reads were assembled using the De Novo Assemble tool implemented in Geneious Prime 2022.2.1 (Biomatters, Ltd., Auckland, New Zealand). Assembled DNA sequences were deposited at GenBank for further studies.

### Sequence alignment, genetic variation, phylogenetic analysis, and network

DNA sequences for ITS and *atp*B-*rbc*L regions were aligned with Clustal Omega 1.2.3 (Sievers and Higgins 2014). To understand the infraspecific genetic variations of *Isnardia* taxa, the nucleotide diversities (Tajima’s π; π) (Tajima 1983) were quantified using MEGA 11 (Tamura et al. 2021) with the Kimura 2-parameter model (Kimura 1980), and 500 replicates were applied to calculate the standard errors. One-way analysis of variance (ANOVA) and student’s t-tests were conducted with SPSS 28.0 (IBM Corp 2021) to test our third hypothesis as well as to assess how sample size affects the genetic diversities of *Isnardia.*

To reconstruct phylogenetic trees and meet our first goal, we applied Maximum likelihood (ML) and Bayesian inference (BI) algorithms. The nucleotide substitution model for each studied region was obtained using jModelTest 2 (Darriba et al. 2012). The ML trees were generated using RAxML 8.2.11 (Stamatakis 2014) with the best-fitting model for each region, and the branch supports were evaluated using the bootstrapping values (bs) (Felsenstein 1985) with 1000 replicates. Bayesian inference (BI) analyses of phylogeny were conducted using MrBayes 3.2.7 (Huelsenbeck and Ronquist 2001; Ronquist et al. 2012) on the CIPRES Science Gateway 3.3 (Miller et al. 2010) with two independent Markov Chain Monte Carlo (MCMC) runs, 5 × 10^6^ generation Markov chains in each run, and the best-fitting models. Trees were saved every 1000 generations. A 50% majority-rule consensus BI tree and the posterior probabilities (pp) on the branches were yielded by incorporating the last 75% of the saved trees. FigTree 1.4.4 (Rambaut 2018) was applied to depict the ML and BI trees.

To infer the evolutionary relationships within *Isnardia*, which includes many allopolyploid taxa, a phylogenetic network was conducted. The biparentally inherited nuclear regions are required in the network analyses to take in both maternal and paternal heritages of allopolyploid organisms. Therefore, a subset of the ITS data was applied here. Based on the *Isnardia* genomes hypothesized by Liu et al. (2020), in the subset, ideally, one sequence would be selected to represent a diploid taxon, two sequences from two different well-supported clades would represent a tetraploid taxon, three sequences from three different well-supported clades would represent a hexaploid, and so on. Here, we randomly selected one sequence to represent a taxon in each well-supported clade from the ITS tree generated above in the present study and made these sequences the subset. The subset was then aligned and analyzed using Clustal Omega 1.2.3 (Sievers and Higgins 2014) and RAxML 8.2.11 (Stamatakis 2014) as described above. The resulting subset ML tree was then converted to a multi-label tree. Subsequently, a phylogenetic network was computed with the multi-label tree algorithm using the Exact Method (Huber et al. 2006) implemented in Dendroscope 3.8.3 (Huson and Scornavacca 2012).

### Divergence time estimation

To achieve our second aim, the age of *Isnardia* TMRCA was estimated using Beast 2.6.7 (Bouckaert et al. 2019) based on the subset ITS data and outgroup sequences from other *Ludwigia* sections and other genera in Onagraceae (see Additional file 1). Sequences were aligned with Clustal Omega 1.2.3 (Sievers and Higgins 2014), and the best-fitting nucleotide substitution model was determined using jModelTest 2 (Darriba et al. 2012). Applying the birth–death skyline model (BDSKY; Stadler et al. 2013), we ran a Markov Chain length of 4 × 10^7^ generations with the estimated best-fitting nucleotide substitution model, substitution rates of ITS in genus *Lopezia* under Onagraceae (5.15 × 10^–9^ substitutions/site/year; O’Kane 1993), and secondary calibration points at the crown node of Onagraceae (71 (88.4–54.3) MYA from Gonçalves et al. (2020) and 46.9 (74.1–2.7) MYA from Zhang et al. (2021)). These two age estimations were both in harmony with those from the fossil records (Grímsson et al. 2011; Lee et al. 2013; Farooqui et al. 2019). Trees were sampled every 1,000 generations. Output log files were checked with Tracer 1.7.1 (Rambaut et al. 2018) to ensure that all Effective Sample Size (ESS) values were greater than 200. The 50% majority-rule consensus trees, pp on the branches, and node heights, or node ages, were produced using TreeAnnotator 2.6.7 (Bouckaert et al. 2019) with a 10% burnin and drawn using FigTree 1.4.4 (Rambaut 2018). Topologies with the uncertainty of the node heights were then generated using DensiTree 2.2.7 (Bouckaert 2010; Bouckaert and Heled 2014).

### IMa analyses

To examine the interspecific hybridization among *Isnardia* taxa as well as to test our fourth hypothesis, we employed the Isolation with Migration coalescent model implemented in IMa2 (Hey and Nielsen 2007) and estimated migration rates in both directions (*m*_0→1_ and *m*_1→0_) between every two *Isnardia* taxa. The substitution rates of chloroplast spacers in seed plants (1.01 × 10^–9^ substitutions/site/year; Graur and Li 2000; Chiang et al. 2006) and nuclear ITS in genus *Lopezia* (5.15 × 10^–9^ substitutions/site/year; O’Kane 1993) were adopted to scale all parameters. Since there were multiple changes at some sites in ITS and *atp*B-*rbc*L regions, the HKY substitution model (Hasegawa et al. 1985) was applied. For each simulation, 1 × 10^7^ generations with 3 × 10^6^ burn-in were performed. Three independent simulations were conducted for every two taxa to assess the consistency among the results. An average of the three results was reported for every two-taxon pair. The migration rate per gene copy per generation (M) was determined using the formula M = *m* × u, where u is the substitution rate per year for the studied region. One-way ANOVA and t-tests were applied to investigate the trends in the interspecific gene flow among ploidy levels in *Isnardia* and test our fourth hypothesis.

## Results

### Sampling, PCR, sequencing, and assembly

Totally, 111 *Isnardia* samples––including 70 newly collected samples, 39 from Hung et al. (2009)’s work, and three from the MO herbarium––were processed. An additional six taxa from other *Ludwigia* sects. and three taxa from other genera in Onagraceae were also included in our analyses. DNA of all newly collected samples and herbarium samples was extracted successfully. Both studied regions were well amplified for most of the newly collected samples. However, after extensive attempts, all PCR failed for the herbarium samples. The herbarium samples were then dismissed from the following analyses. No multiple sizes were found in PCR products, and no polymorphism base was recognized in the assembled sequences. Therefore, cloning was not conducted. The voucher information and GenBank accession numbers of all studied samples are provided in Additional file 1.

### Phylogenetic analysis, network, and genetic variation

In total, 192 sequences representing 20 of 22 *Isnardia* taxa and ten sequences representing nine outgroups were analyzed in the present study, within which 124 *Isnardia* sequences were newly generated (Table 1; Additional file 1).

**Fig. 1:**
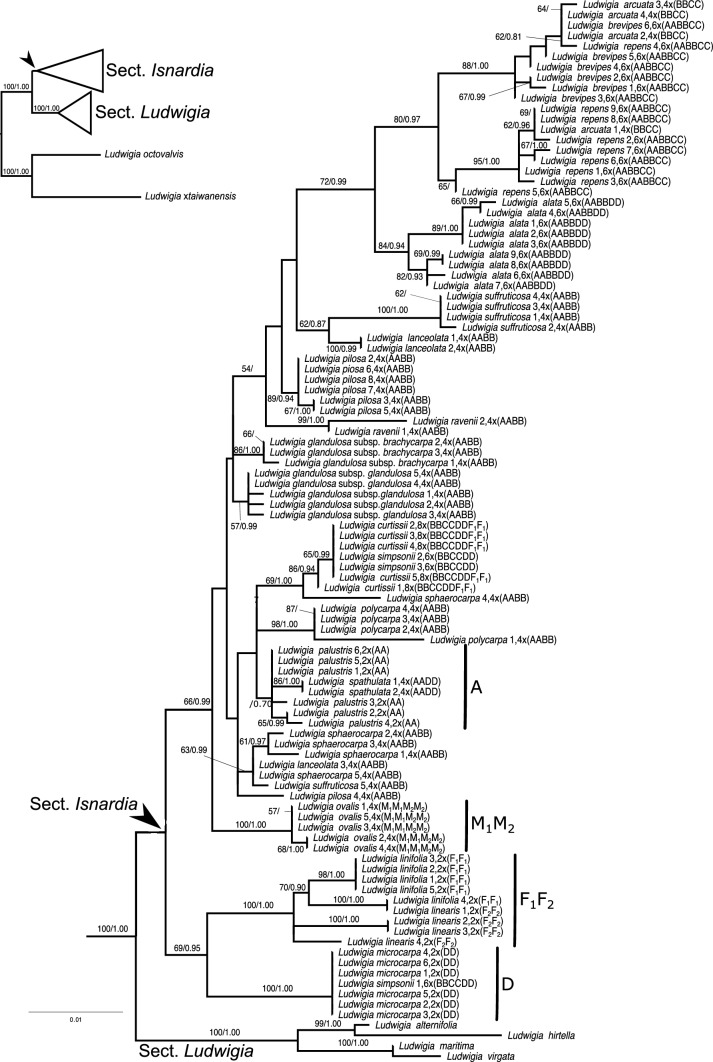
Maximum likelihood tree of *Ludwigia* sect. *Isnardia* inferred from the nuclear ITS region. The arrows indicate the crown nodes of *Isnardia*. Sample numbers (Additional file 1), ploidy levels (Table 1), and genome types (Table 1) are shown right after the taxa. Numbers at nodes show the bootstrapping values (bs) / posterior probabilities (pp) only when the bs at nodes are greater than 50 or pp at nodes are greater than 0.70. The A, M_1_M_2_, F_1_F_2_, and D clades are indicated. The scale bar denotes the branch length

**Fig. 2:**
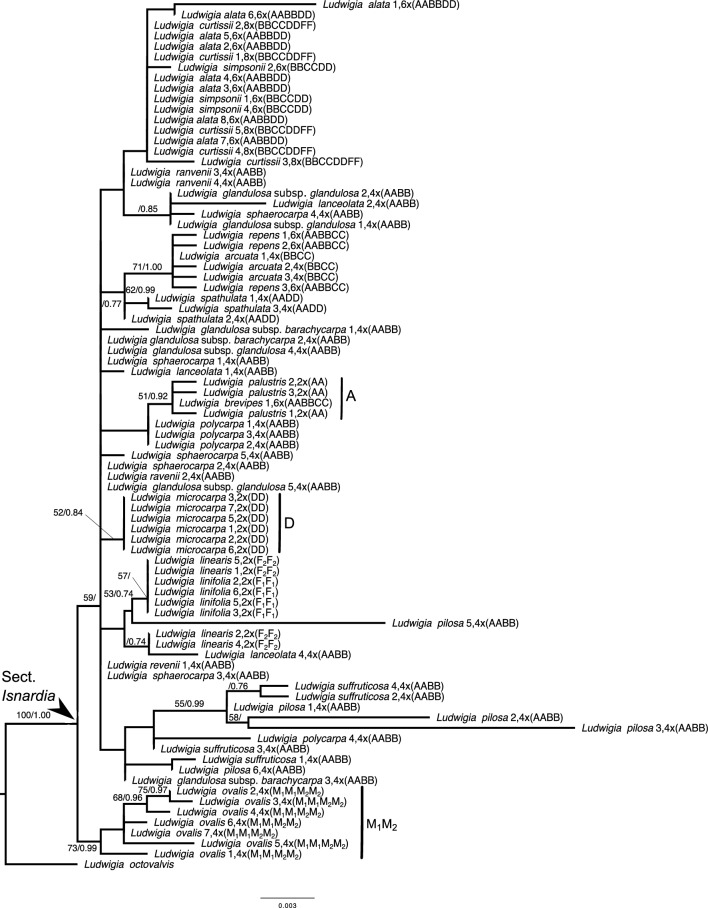
Maximum likelihood tree of *Ludwigia* sect. *Isnardia* inferred from the chloroplast *atp*B-*rbc*L region. The arrow indicates the crown node of *Isnardia*. Sample numbers (Additional file 1), ploidy levels (Table 1), and genome types (Table 1) are shown right after the taxa. Numbers at nodes show the bootstrapping values (bs) / posterior probabilities (pp) only when the bs at nodes are greater than 50 or pp at nodes are greater than 0.70. The scale bar denotes the branch length

Both ML and BI trees were reconstructed for *atp*B-*rbc*L and ITS regions. The best-fitting substitution models and descriptive statistics of the two studied regions are shown in Table 2. The alignments and tree files are provided in Additional file 2 and Additional file 3. Since BI trees share most topologies with ML trees, only ML trees are shown here (Figs. 1 & 2). Our ITS tree (Fig. 1) was highly consistent with nuclear trees of Hung et al. (2009) and Liu et al. (2017). However, *Isnardia* taxa were clustered together with weak support in our ITS tree (pp = 0.66, bs = 44; Additional file 2 & Additional file 3). The well-supported clades revealed in the ITS tree were used to set up the subset ITS data (see network analyses). Adopting the hypothesized genome types of *Isnardia* inferred by Liu et al. (2020) (Table 1), we identified the A, M_1_M_2_, F_1_F_2_, and D clades (Fig. 1). For example, the A clade comprised the diploid *L. palustris* and all other samples which had A in their genome types (Fig. 1). Though the resolutions of our *atp*B-*rbc*L tree (Fig. 2) among all other *Isnardia* were generally low, our tree was largely congruent with the chloroplast phylogenies of Hung et al. (2009) and Liu et al. (2017, 2020), and *L. ovalis* Miq. was sister to all other *Isnardia* taxa.

**Table 2:** The substitution models, characteristics, and maximum parsimony statistics of the regions

Region	Substitution model	Number of sequences	Number of outgroup sequences	Consistency index	Retention index	Parsimony-informative characters/ Total characters (%)
ITS	GTR + G	104	6	0.7399	0.9189	111/662 (16.77%)
*atp*B-*rbc*L	GTR + I + G	83	1	0.8393	0.8636	28/850 (3.29%)

Moreover, our phylogenetic analyses showed that both tetraploid *L. sphaerocarpa* (AABB) and hexaploid *L. repens* (AABBCC) have evolved multiple times, and tetraploid *L. arcuata* Walter (BBCC) might have multiple origins or a higher infraspecific genetic variation. For *L. sphaerocarpa*, one evolutionary scenario showed that the paternal and/or maternal donors of *L. sphaerocarpa* samples 1–3 and 5 were phylogenetically close to *L. lanceolata* Elliott (AABB) and *L. suffruticosa* Walter (AABB), two members of the AABB tetraploids (Fig. 1; Table 1; Additional file 1). This evolutionary scenario might fit earlier hypotheses on the homoploid hybridization of *L. sphaerocarpa* (Peng 1988; Liu et al. 2020) if our *atp*B-*rbc*L tree (Fig. 2) had higher resolution and gave more information on the maternal donors of these four *L. sphaerocarpa* samples. We detected another evolutionary scenario that revealed that the maternal donor of *L. sphaerocarpa* sample 4 probably carried genome A and was likely closely related to *L. lanceolata* and *L. glandulosa* Walter subsp. *glandulosa* (AABB), which both had an AABB genome type (Fig. 2; Table 1). The paternal donor contributing genome B to this *L. sphaerocarpa* sample likely also gave rise to *L. curtissii* Chapm. (BBCCDDF_1_F_1_) and *L. simpsonii* Chapm*.* (BBCCDD) (Fig. 1; Table 1). The latter evolutionary scenario of *L. sphaerocarpa* has not been revealed by earlier studies.

For the origins of *L. repens*, our *atp*B-*rbc*L tree (Fig. 2) suggests that the maternal donors were probably tetraploids carrying genome BBCC and closely related to *L. arcuata*. This result was congruent with earlier studies (Hung et al. 2009; Liu et al. 2020). Moreover, our ITS tree (Fig. 1) indicated that at least two ancestor lineages––likely the maternal donors with genome BBCC––had given birth to *L. repens*; one gave rise to *L. repens* sample 4 while another contributed to *L. repens* samples 1–3 and 5–9. The former maternal donor also played a part in the origination of *L. brevipes* (Long) Eame (AABBCC). However, no paternal donor of *L. repens* with genome A was represented in our study.

Our ITS tree shows that *L. arcuata* samples are clustered in two well-supported groups (Fig. 1). One group consists of *L. arcuata* samples 2–4, *L. brevipes* samples 1–6, and *L. repens* sample 4, while another group includes *L. arcuata* sample 1 and *L. repens* samples 1–3 and 5–9. This indicates that *L. arcuata* probably has multiple origins and/or a relatively high infraspecific nucleotide diversity. In fact, our analyses show that the infraspecific π of *L. arcuata* was higher than that of most *Isnardia* (Table 1).

**Fig. 3:**
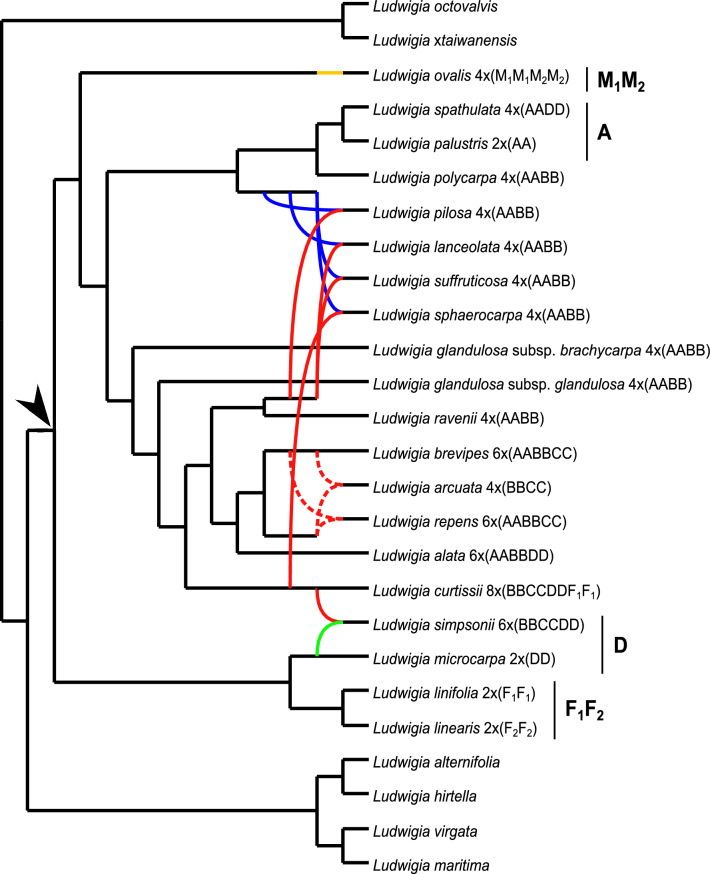
Evolutionary network of *Ludwigia* sect. *Isnardia* inferred from the subset of the ITS region. The arrow indicates the crown node of *Isnardia*. The A, M_1_M_2_, F_1_F_2_, and D clades are marked as in Fig. 1. Blue lines show the evolutionary signal from the A genome, red lines from the B genome, dashed red lines likely from the B genome, green lines from the D genome, brown lines from the M_1_ and M_2_ genomes

Twenty-nine *Isnardia* samples were included in the subset ITS data and network analyses. Sample information of the subset is provided in Additional file 1. The phylogenetic network (Fig. 3) concurs with our ITS tree (Fig. 1) completely and indicates the hybrid origins of eight *Isnardia* taxa. Tetraploids *L. lanceolata*, *L. pilosa* Walter, and *L. suffruticosa* share the same evolutionary histories, and all of them are allopolyploids deriving from two genomes (Fig. 3). One evolutionary signal arose from the lineage sister to the clade/genome A. Another signal is likely from genome B because this signal arose from the lineage sister to *L. ravenii* C.I Peng, which has B in its genome type. Our ITS and *atp*B-*rbc*L trees (Figs. 1, 2) indicate that tetraploid *L. sphaerocarpa* had evolved at least twice (see previous paragraphs). The first evolutionary scenario, homoploid hybridization (Peng 1988; Liu et al. 2020), could not be shown in the network due to the limitation of our sampling. The second evolutionary scenario is revealed in our network (Fig. 3); that *L. sphaerocarpa* obtained genome A from the lineage sister to the A clade/genome and obtained its genome B from *L. curtissii*, which also has genome B. Moreover, two evolutionary signals were detected for the origins of tetraploid *L. arcuata* and hexaploid *L. repens*, and these two evolutionary signals were from sister lineages, which both lineages might have genome B (dashed red lines in Fig. 3). This may indicate that both *L. arcuata* and *L. repens* have multiple origins. Furthermore, our network implies that hexaploid *L. simpsonii* stemmed from at least two genomes––D genome from the lineage close to diploid *L. microcarpa* and B genome from the lineage close to *L. curtissii*. In addition, two evolutionary signals were mixed to give rise to *L. ovalis*. However, it was difficult to distinguish M_1_ and M_2_ signals in our network. Alignment, the ML tree, and network files are given in Additional file 4.

**Fig. 4:**
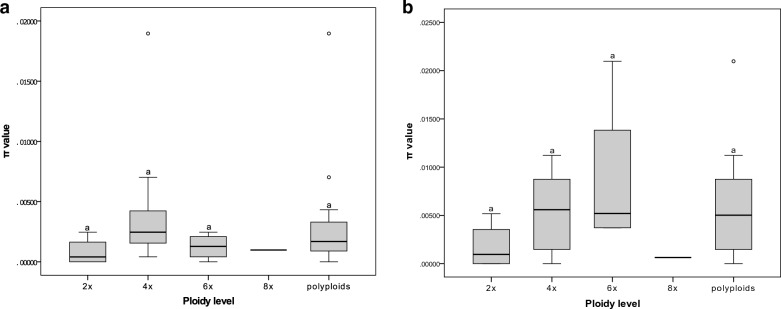
Boxplots showing infraspecific nucleotide diversities (Tajima’s π) of different ploidy levels in *Ludwigia* sect. *Isnardia* based on (**a**) *atp*B-*rbc*L and (**b**) ITS data. Letters above each boxplot indicate pairwise statistical differences between ploidy levels. The complete details for the statistical analysis are shown in Additional file 5. The infraspecific nucleotide diversity of each taxon is available in Table 1

Furthermore, the results of our genetic variation analyses of *Isnardia* suggest that the *atp*B-*rbc*L region generally has lower infraspecific genetic variations (π = 0.00000–0.01896) compared to the ITS region (π = 0.00000–0.02097). The infraspecific π of each *Isnardia* taxon are shown in Table 1. Notably, ITS sequences are infraspecific identical in *L. microcarpa* Michx. and *L. spathulata* Torr. & A. Gray, and *atp*B-*rbc*L sequences are infraspecific identical in *L. linifolia* Poir. and *L. microcarpa*. One-way ANOVA analyses showed that the genetic diversities of *Isnardia* taxa had no significant differences among sample sizes (p-values > 0.05; Additional file 5). In other words, our sampling was appropriate for assessing the genetic diversities in *Isnardia*. Furthermore, our study detected no relationship between ploidy levels and genetic diversities (Fig. 4). For instance, the π value of the ITS region for diploid *L. palustris* (AA) was 0.00189 ± 0.00115, but some of the tetraploids with the AABB genome type had lower π values and others had higher π values (Table 1). One-way ANOVA analyses indicated no significant differences in π values of *Isnardia* taxa among ploidy levels (p-values > 0.05; Fig. 4; Additional file 5). Student’s t-tests also showed no significant differences in π values between diploids and all polyploids (p-values = 0.81 and 0.08 for ITS and *atp*B-*rbc*L regions, respectively). That is, our results falsified hypothesis three.

### Divergence time estimation

**Fig. 5:**
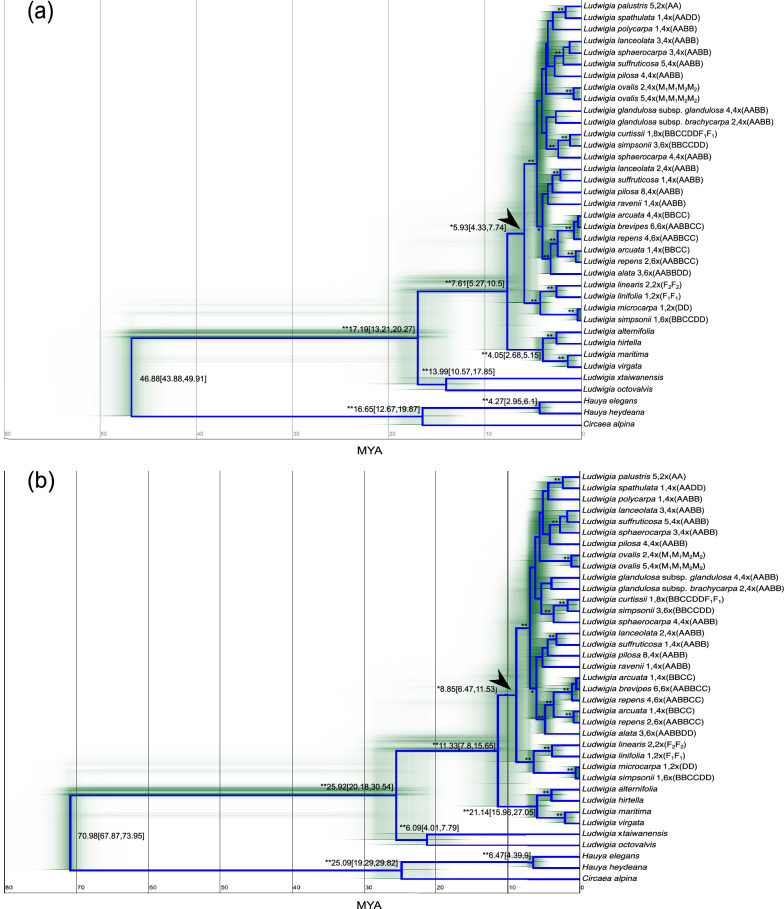
Chronograms of *Ludwigia* sect. *Isnardia* based on the subset of ITS region with divergence time estimates adopting the substitution rates of ITS (O’Kane 1993) and secondary calibration point at the crown node of Onagraceae following (**a**) Zhang et al. (2021) or (**b**) Gonçalves et al. (2020). The arrows indicate the crown nodes of *Isnardia*. Sample numbers (Additional file 1), ploidy levels (Table 1), and genome types (Table 1) are shown right after the taxa. Stars at nodes indicate the posterior probabilities (pp) only if the pp at the nodes is greater than 0.90. When the pp at the nodes is greater than 0.95, the double star notations are denoted. Numbers at nodes show the median estimated divergence times (MYA) with 95% credible intervals in the brackets. The smears around the nodes display the uncertainty of the estimated divergence times

All (29) *Isnardia* samples in the subset and nine outgroups were applied to estimate the age of *Isnardia* TMRCA*.* Sample information is provided in Additional file 1. The topologies of the resulted chronograms (Fig. 5) are congruent with our ITS tree (Fig. 1). Adopting the secondary calibration points at the crown node of Onagraceae suggested by Zhang et al. (2021), the crown node of genus *Ludwigia* was approximately 17.2 (20.3–13.2) MYA, the crown node of the North Temperate Haplostemonous (NTH) *Ludwigia* (= sects. *Isnardia* + *Ludwigia*) was 7.6 (10.5–5.3) MYA, and TMRCA of *Isnardia* was 5.9 (7.7–4.3) MYA (Fig. 5a). When we took Gonçalves et al. (2020)’s results as calibration points instead, the crown node of genus *Ludwigia* was estimated to be 25.9 (30.5–20.2) MYA, the crown node of the NTH *Ludwigia* was 11.3 (15.7–7.8) MYA, and TMRCA of *Isnardia* was 8.9 (11.5–6.5) MYA (Fig. 5b). Alignment and 50% majority-rule consensus tree files are shown in Additional file 6.

### IMa analyses

**Fig. 6:**
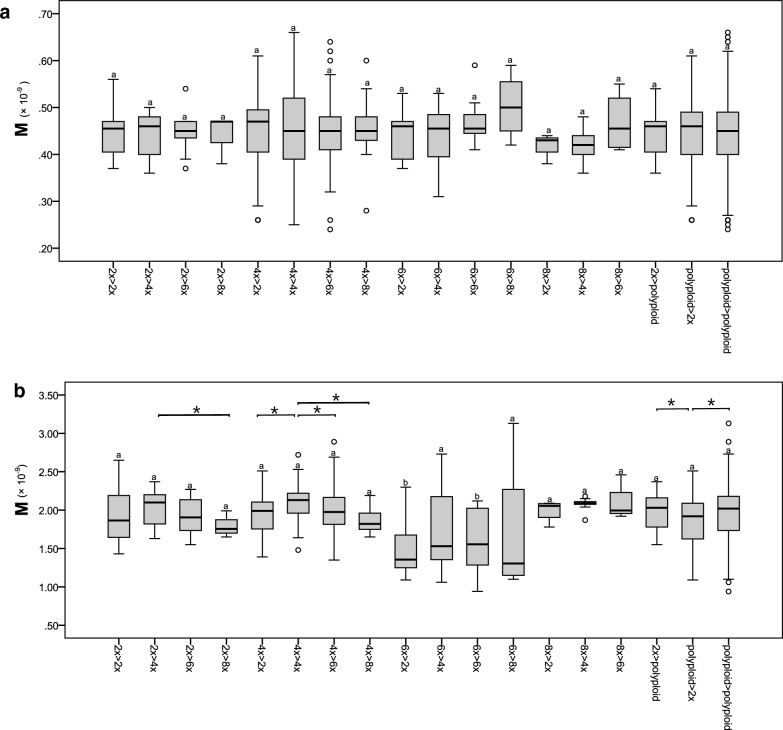
Boxplots showing the estimated migration rates (M, migration rates per gene copy per generation) between ploidy levels in *Ludwigia* sect. *Isnardia* based on (**a**) *atp*B-*rbc*L and (**b**) ITS data. Groups on the x axial are coded, showing the direction of the migration rates, e.g., 2 × > 4 × indicates the migration rates from diploids to tetraploids. Letters above each boxplot indicate pairwise statistical differences (p-values < 0.05) between 2 × > 2 × and other groups. The asterisks indicate pairwise statistical differences (p-values < 0.05) between the two groups. The complete details for the statistical analysis are shown in Additional file 8. The estimated migration rates among taxa are available in Additional file 7

The migration rates per gene copy per generation (M, interspecific gene flow) for every two *Isnardia* taxa based on ITS and *atp*B-*rbc*L regions revealed rampant but low interspecific gene flow in *Isnardia*, from 9.42 × 10^–10^ to 3.13 × 10^–9^, and 2.36 × 10^–10^ to 6.58 × 10^–10^, respectively (Fig. 6, Additional file 7). Moreover, our ANOVA and t-tests showed no significant differences in M among ploidy levels based on *atp*B-*rbc*L data but found some significant differences in ITS data (Fig. 6, Additional file 8). The M between two ploidy levels––except the M from hexaploids to diploids (group 6 × > 2 ×) and from hexaploids to octoploids (group 6 × > 8 ×) based on ITS data––were compatible with the M between two diploids (group 2 × > 2 ×) (see Fig. 6, Additional file 8). Moreover, Fig. 6b shows that the means of Ms from hexaploids to other ploids (including groups 6 × > 2 × , 6 × > 4 × , 6 × > 6 × , and 6 × > 8 ×) were lower than the means of Ms of other groups. Our data supported hypothesis four and indicated that Ms from hexaploids to other taxa were relatively lower.

Furthermore, unsymmetrical Ms were detected in most two-taxon pairs (Additional file 7), and ITS data gave more information on the natural dynamic in *Isnardia* (Fig. 6b, Additional file 8a). Both the M of group 2 × > polyploid were significantly greater than the M of group polyploid > 2 × . In addition, our ITS data indicated that the M of group 4 × > 4 × was considerably higher than the Ms of groups 4 × > 2 × , 4 × > 6 × , and 4 × > 8 × , and hexploids had the same trend but no statistical significance. Diploids, however, showed an opposite trend without statistical significance. The M of group 2 × > 2 × was lower than the Ms of groups 2 × > 4 × and 2 × > 6 × , but higher than the M of group 2 × > 8 × .

## Discussions

With an extensive sampling of *Isnardia* (Table 1; Additional file 1), we met our four aims of this investigation of the polyploid clade’s evolutionary history and dynamic nature.

### Evolutionary relationships and polyploid speciation in *Isnardia*

Incorporating the evolutionary information provided by our phylogenetic trees and network, our data fully support the reticulate evolution and hypotheses as to the genome types of *Isnardia* taxa indicated by Peng (1989), Peng et al. (1988, 2005), and Liu et al. (2020), despite the generally low resolutions in our *atp*B-*rbc*L tree (Fig. 2) and low supports at several basal branches in our ITS tree (Fig. 1). Low resolutions of the chloroplast trees inhibited the understanding of *Isnardia* evolutionary history in earlier studies (Hung et al. 2009; Liu et al. 2017, 2020) and in ours. Some recent studies on other *Ludwigia* clade and other plant groups (e.g. Liu et al. 2018; Duvall et al. 2020; Simmonds et al. 2021) have shown that the next-generation sequencing (NGS) data would provide complete plastome sequences and improve the understanding of maternal evolutionary inferences. Further studies with NGS data will be called on to uncover maternal phylogeny in *Isnardia* thoroughly. Additional nuclear single-copy genes may be needed for the phylogenetic network of *Isnardia* to comprehend reticulate evolution within this polyploid section (e.g. Díaz-Pérez et al. 2018; Wang et al. 2019; Karbstein et al. 2022).

Moreover, we recognized the A, M_1_M_2_, F_1_F_2_, and D clades but could not identify the B and C clades (Figs. 1, 2, 3). Very likely, diploids of the latter two clades were extinct (Liu et al. 2020). Interpreting the evolutionary history of polyploid groups without their diploid ancestors is challenging (Holloway et al. 2006; Yuan et al. 2006; Soltis and Soltis 2016). The NGS data and advanced algorithms may be helpful in further evolutionary studies on polyploid groups with extinct diploid progenitors, like *Isnardia* (e.g. Li et al. 2022; Sancho et al. 2022).

Furthermore, our analyses unveiled the complexity of polyploid speciation in *Isnardia*, which has not been reported before. Our study revealed that *L. repens* has evolved at least twice (Figs. 1, 2, 3), which supports the work of Liu et al. (2020). Moreover, based on our data, the multiple origins of *L. arcuata* are suggested here for the first time (Figs. 1, 2, 3). In addition, our data indicate that *L. sphaerocarpa* arose in various ways (Figs. 1, 2, 3). As per previous cytological and molecular studies, *L. sphaerocarpa* has occurred multiple times through the homoploid hybridization between two tetraploid ancestors, which both have an AABB genome type (Peng 1988; Liu et al. 2020). The homoploid hybridization proposition is also supported by our *L. sphaerocarpa* samples 1–3, and 5 (Figs. 1, 2). Moreover, our *L. sphaerocarpa* sample 4 uncovers an additional evolutionary scenario showing that genomes A and B were contributed from different lineages (Figs. 1, 2). Unfortunately, the genome types and ploidy levels of maternal and paternal donors of *L. sphaerocarpa* sample 4 are still unknown because of our limited data. This additional evolutionary scenario not only highlights the complexity of allopolyploidization in *L. sphaerocarpa* but also offers another route to explore the unidentified or extinct diploid ancestors with genome B. With the application of NGS data, further studies may sample more individuals from polyploid *Isnardia* taxa with genome B and track back how genome B had contributed to polyploid speciation in *Isnardia* (e.g. Li et al. 2022; Sancho et al. 2022)*.*

Additionally, we sampled herbarium *L. stricta*, a Cuban endemic, but obtained no PCR result. However, this was not unexpected and may be attributed to the degraded DNA of herbarium vouchers. *Ludwigia stricta* is a diploid, morphologically similar to *L. linifolia* (Peng and Tobe 1987; Peng 1988, 1989), and may be valuable for understanding the reticulate evolution of *Isnardia*. Some NGS strategies have successfully obtained ample sequences from herbarium samples of various plant groups (e.g. Vatanparast 2018; Couvreur et al. 2019; Vargas et al. 2019) and their use should help obtain evolutionary information from *L. stricta* and other *Isnardia* herbarium vouchers.

### Divergence time estimation

With a more comprehensive sampling in *Isnardia* (Table 1 and Additional file 1) and using the secondary calibrations at Onagraceae crown nodes from recent studies (Gonçalves et al. 2020; Zhang et al. 2021), our analyses showed that the ages of *Isnardia* TMRCA were 5.9 (7.7–4.3) MYA and 8.9 (11.5–6.5) MYA (see Fig. 5), which are in concordance with Hung et al. (2009)’s study (6.59 ± 0.02 MYA), and are younger than the fossil record (ca. 11.63–15.97 MYA, see Friis 1985; Tobe et al. 1988). Many plant and animal study cases have reported incongruences between molecular and fossil dates (Benton and Ayala 2003; Heads 2005). Our case is likely due to the extensive polyploidization in *Isnardia*. The molecular date estimate based on one paralogue––which is equal to the genome presented in the current study––is theoretically younger than the date of the gene duplication, and all paralogues are requisite to better determine the gene duplication date (Jiao et al. 2011; Ruprecht et al. 2017; Koenen et al. 2021). Unfortunately, as shown in the Results section and Figs. 1 and 3, not all genomes were sequenced for all *Isnardia* polyploids. This probably led to the finding of a younger age of *Isnardia* TMRCA by our analyses. Nonetheless, other factors causing inconsistency between the estimated *Isnardia* TMRCA and fossil records (like inaccurate substitution rate, extinct/unsampled taxa, and unoptimistic algorithm) cannot be ruled out (Linder et al. 2005; Doyle and Egan 2010; Guindon 2020). Obtaining improved divergence time estimates for *Isnardia* in further studies would not be challenging without all genome/paralogues of single-copy genes, comprehensive taxa sampling, and extensive analyses.

### Dynamic nature of *Isnardia*––infraspecific genetic diversity and interspecific gene flow

Infraspecific genetic diversity reflects the evolutionary history of a taxon, including origin, historical events, life history traits, and geographical distribution pattern. (Avise 2000; Sweigart and Willis 2003; Mallet 2007; Bogačiovienė et al. 2019). As revealed in an earlier study on six *Isnardia* taxa (Hung et al. 2009), our data also show that the infraspecific nucleotide diversities (π) of *Isnardia* are generally lower in the chloroplast *atp*B-*rbc*L region than in the nuclear ITS region (Table 1). This has been exhibited in many other plant groups and attributed to the chloroplast sequences being more conserved compared to nuclear sequences in plants (e.g. Perdereau et al. 2014; Xu et al. 2015; Li et al. 2018).

Moreover, our data indicate that three *Isnardia* taxa––*L. linifolia*, *L. microcarpa*, and *L. spathulata*––have infraspecific identical sequences in the *atp*B-*rbc*L or ITS regions (Table 1). None or low infraspecific genetic variation may indicate short evolutionary time, reproductive strategies leading to genetic homogeneity (e.g. Bussell 1999; Sweigart and Willis 2003; Duffy et al. 2009), evolutionary historical events resulting in population size reductions (e.g. Pimm et al. 1989; Ellegren and Galtier 2016), or evolutionary constraints (e.g. Futuyma 2010; Baucom 2019) of the taxon. These three *Isnardia* taxa had arisen at least 0.30 or 0.44 MYA (Fig. 5a or b; Additional file 6), which should be long enough to accumulate some infraspecific genetic variation. None or low genetic variations in these three taxa are unlikely to be ascribable to the short evolutionary time. Furthermore, early systematic studies have reported that *L. microcarpa* and *L. spathulata* are facultatively autogamous, while *L. linifolia* is an outcrossing taxon (Raven and Tai 1979; Peng 1988, 1989; Peng et al. 2005; Wagner et al. 2007). Autogamy might cause none or low infraspecific genetic variation in *L. microcarpa* and *L. spathulata*. In contrast, the mating system of *L. linifolia* contributes little to its infraspecific identical sequence. Furthermore, other plant groups in eastern North America had suffered population shrinkage resulting from climate changes (e.g. Woodruff et al. 1981; Zubakov and Borzenkova 1990; Groot 1991) or habitat destruction (e.g. Vargas-Rodriguez et al. 2015; Mohn et al. 2021; Ony et al. 2021). The three *Isnardia* taxa may also reduce their population sizes under such environmental disturbances. Additionally, having a restricted distribution is one of the common characteristics of a taxon under evolutionary constraints (Futuyma 2010; Razgour et al. 2019). No genetic variation in *L. spathulata*, a rare species (Peng et al. 2005), might be credited to the evolutionary constraints of this taxon. However, *L. microcarpa* and *L. linifolia* are relatively common (Peng 1988, 1989), and some other causes might apply. Additional sampling and analysis are required to elucidate the taxa without infraspecific genetic variations.

In addition, many earlier studies focusing on infraspecific ploidy taxon or a small polyploid complex with a few taxa unveiled that higher infraspecific genetic diversities were found in taxa with higher ploidy levels (e.g. Mallet 2007; García‐Verdugo et al. 2009; Bogačiovienė et al. 2019; Zhang et al. 2019). We expected to find the same pattern in the massive polyploid complex, *Isnardia* (hypothesis three). Surprisingly, our data did not support this hypothesis (Table 1; Fig. 4; Additional file 5). This unanticipated result may be attributed to the long history of *Isnardia* taxa (Fig. 5). Each taxon may undergo different evolutionary events, diluting the effect of ploidy levels on infraspecific genetic diversity (e.g. Riddle et al. 2006; García‐Verdugo et al. 2009). The studied taxa's evolutionary and ecological attributes may also blur this pattern. Apart from the mating systems and evolutionary constraints mentioned in the earlier discussions, other attributes like geographic environments and climate factors were reported to affect infraspecific genetic diversity significantly in polyploid groups (e.g. Zhang et al. 2017; Ahrens et al. 2020; Androsiuk et al. 2021; Tsuruta et al. 2022). Further studies will be required to explain the lack of an effect of ploidy level on infragenetic genetic diversity in *Isnardia*.

Furthermore, our IMa analyses reveal the prevalent but low interspecific gene flow among *Isnardia* taxa (Fig. 6, Additional files 7 and 8) and provide a solid genetic basis for the rampant interspecific hybridization in *Isnardia* reported by earlier field observations and breeding experiments (Raven and Tai 1979; Peng 1988, 1989; Peng et al. 2005). Polyploidization is considered one of the important speciation mechanisms in plants, and prezygotic and/or postzygotic reproductive barriers between ploidy levels are expected. However, an increasing number of studies have reported that polyploidization is extremely complicated (Ramsey and Schemske 1998; Husband and Sabara 2004; Baack et al. 2015) and further suggested that polyploidization in some plant groups could collapse the reproductive barriers among ploidy levels and potentiate interspecific gene flow (e.g. Jørgensen et al. 2011; Bohutínská et al. 2021; Schmickl and Yant 2021). While most studies focused on autopolyploidization-mediated interspecific gene flow and its mechanisms (e.g. Husband and Sabara 2004; Przewieslik-Allen et al. 2021; Schmickl and Yant 2021), only a few reported that allopolyploidization may also break down the reproductive barriers among ploidy levels (e.g. Iqbal et al. 2019). *Isnardia* is the first plant group with frequent interspecific gene flows among multiple allopolyploids and diploids supported by field observations, greenhouse breeding experiments, and genetic data.

Additionally, our data suggest unsymmetrical interspecific gene flows between every two taxa and two ploidy levels in *Isnardia* (Fig. 6, Additional files 7 and 8); however, this phenomenon was not detected in earlier *Isnardia* studies (Peng 1988, 1989; Peng et al. 2005). Previous reports have observed unsymmetrical interspecific gene flows in many plant groups (e.g. Peng and Chiang 2000; Lafon-Placette et al. 2017; Abdelaziz et al. 2021). Both prezygotic and postzygotic reproductive barriers could contribute to this phenomenon (Rahmé et al. 2009; Zhang et al. 2016; Moreira-Hernández and Muchhala 2019). Further studies with genome-wide genetic information and breeding experiments with cytological work may provide more insights into the mechanisms of weakening reproduction barriers among allopolyploids and diploids and asymmetric interspecific gene flow in *Isnardia*.

## Conclusions

In summary, a comprehensive sampling of *Ludwigia* sect. *Isnardia*, an allopolyploid complex, was applied to test four hypotheses. First, our phylogenetic trees and network not only support the earlier reticulate evolution hypotheses and proposed genomes but also recognize three multiple-origin taxa––tetraploid *L. arcuata*, hexaploid *L. repens*, and tetraploid *L. sphaerocarpa*. The multiple origins of *L. arcuata* and a new evolutionary scenario of *L. sphaerocarpa* are reported in this study for the first time. Second, our age estimates of *Isnardia* TMRCA (5.9 or 8.9 MYA) align with the earlier estimations but are younger than fossil records. In future studies, sampling all paralogues of the studied DNA regions of all taxa would yield a better TMRCA estimate for a polyploid complex like *Isnardia*. Our analyses did not support hypothesis three, that higher infraspecific genetic variations would be found in taxa with higher ploidy levels. The long evolutionary history and ecological characteristics may have influenced the infraspecific genetic variation of an *Isnardia* taxon. In addition, we observed the prevailing and unsymmetrical interspecific gene flows among *Isnardia* taxa, which suggests that the reproductive barriers among taxa were reduced. This has rarely been reported in an allopolyploid complex and calls for additional research. Taken together, our study provides several new insights into the evolutionary history and dynamic nature of *Isnardia* and highlights the need for further studies to understand the allopolyploid complex.

## Supplementary Information

**Additional file 1.** Samples applied in this study, their GenBank accession numbers, and voucher information.

**Additional file 2.** Alignment, ML tree, and BI tree files of ITS region.

**Additional file 3.** Alignment, ML tree, and BI tree files of the *atp*B-*rbc*L region.

**Additional file 4.** Alignment, ML tree, and network files of the subset ITS data.

**Additional file 5.** One-way ANOVA analyses and student’s t-tests show that the genetic diversities of *Isnardia* taxa had no significant differences among ploidy levels and sample sizes.

**Additional file 6.** Alignment and consensus tree files for divergence time estimates.

**Additional file 7.** The estimated migration rates of every two taxa in *Ludwigia* sect. *Isnardia.*

**Additional file 8.** One-way ANOVA analyses and student’s t-tests for the estimated migration rates between two ploidy levels in *Ludwigia* sect. *Isnardia.*

## Data Availability

All data generated and analyzed during this study are included in this published article and its additional information files.

## Abbreviations

BDSKY: Birth–death skyline model
BI: Bayesian inference
bs: Bootstrapping value
ESS: Effective sample size
M: Migration rates per gene copy per generation
ML: Maximum likelihood
MYA: Million years ago
pp: Posterior probability
TMRCA: The most recent common ancestor
