# Supplementary material for: New insights into polyploid evolution and dynamic nature of Ludwigia section Isnardia (Onagraceae)
Source: Bot Stud. 2023 Jun 3;64:14. doi: 10.1186/s40529-023-00387-8 (PMC10239408; doi:10.1186/s40529-023-00387-8)
Supplement: Supplementary file 1 — Additional file 1. Samples applied in this study, their GenBank accession numbers, and voucher information. [file 40529_2023_387_MOESM1_ESM.docx]

**Additional File 1.** Samples applied in this study, their GenBank accession numbers, and voucher information. The sequences indicated with asterisks are newly generated, and others were obtained from GenBank. The em dashes indicate missing data.

| **Taxon** | **Locality** | **Collector and collection number** | **ITS** | ***atp*B-*rbc*L** |
| --- | --- | --- | --- | --- |
| **Outgroups** |  |  |  |  |
| *Hauya elegans* DC. |  |  | AY357767 | -- |
| *Hauya heydeana* Donn. Sm. |  |  | GQ232550 | -- |
| *Circaea alpina* L. |  |  | MH808731 | -- |
| *Ludwigia alternifolia* L. |  |  | KX168309 | -- |
| *Ludwigia hirtella* Raf. |  |  | KX168327 | -- |
| *Ludwigia maritima* R.M. Harper |  |  | KX168344 | -- |
| *Ludwigia octovalvis* (Jacq.) P.H. Raven |  |  | KX168358 | KX827312 |
| *Ludwigia* ×*taiwanensis* C.-I Peng |  |  | KX168387 | -- |
| *Ludwigia virgata* Michx. |  |  | KX168385 | -- |
| **Ingroups** |  |  |  |  |
| *Ludwigia alata* Elliott 1 | USA, Florida, Levy Co. | Tsai-Wen Hsu 11279 | OK338626* | OK381697* |
| *Ludwigia alata* Elliott 2 | USA, Florida, Citrus Co. | Tsai-Wen Hsu 11281 | OK338568* | OK381702* |
| *Ludwigia alata* Elliott 3 | USA, Florida, Hernando Co. | Tsai-Wen Hsu 11283 | OK338569* | OK381701* |
| *Ludwigia alata* Elliott 4 | USA, Florida, Putnum Co. | Tsai-Wen Hsu 11350 | OK338570* | OK381699* |
| *Ludwigia alata* Elliott 5 | USA, Florida, Volusia Co. | Tsai-Wen Hsu 11346 | OK338571* | OK381700* |
| *Ludwigia alata* Elliott 6 | USA, Florida, Gulf Co. | Tsai-Wen Hsu 11256 | OK338572* | OK381705* |
| *Ludwigia alata* Elliott 7 | USA, Florida, Gulf Co. | Tsai-Wen Hsu 11256 | OK338573* | OK381714* |
| *Ludwigia alata* Elliott 8 | USA, Florida, Gulf Co. | Tsai-Wen Hsu 11257 | OK338574* | OK381715* |
| *Ludwigia alata* Elliott 9 | USA, Florida, Gulf Co. | Tsai-Wen Hsu 11257 | OK338575* | -- |
| *Ludwigia arcuata* Walter 1 |  |  | FN263223 | FN263195 |
| *Ludwigia arcuata* Walter 2 |  |  | FN263226 | FN263196 |
| *Ludwigia arcuata* Walter 3 |  |  | FN263227 | FN263197 |
| *Ludwigia arcuata* Walter 4 |  |  | KX168310 | -- |
| *Ludwigia brevipes* (Long) Eame 1 |  |  | FN263235 | FN263191 |
| *Ludwigia brevipes* (Long) Eame 2 |  |  | FN263236 | -- |
| *Ludwigia brevipes* (Long) Eame 3 |  |  | FN263237 | -- |
| *Ludwigia brevipes* (Long) Eame 4 |  |  | FN263238 | -- |
| *Ludwigia brevipes* (Long) Eame 5 |  |  | FN263239 | -- |
| *Ludwigia brevipes* (Long) Eame 6 |  |  | KX168313 | -- |
| *Ludwigia curtissii* Chapm. 1 | USA, Florida, Manatee Co. | Tsai-Wen Hsu 11292 | OK338612* | OK381720* |
| *Ludwigia curtissii* Chapm. 2 | USA, Florida, Gladeo Co. | Tsai-Wen Hsu 11316 | OK338610* | OK381698* |
| *Ludwigia curtissii* Chapm. 3 | USA, Florida, Collier Co. | Tsai-Wen Hsu 11323 | OK338608* | OK381719* |
| *Ludwigia curtissii* Chapm. 4 | USA, Florida, Okechobee Co. | Tsai-Wen Hsu 11333 | OK338609* | OK381721* |
| *Ludwigia curtissii* Chapm. 5 | USA, Florida, Seminole Co. | Tsai-Wen Hsu 11340 | OK338611* | OK381723* |
| *Ludwigia glandulosa* Walter subsp. *glandulosa* 1 | USA, Tennessee, Dyer Co. | Tsai-Wen Hsu 11370 | OK338564* | OK381716* |
| *Ludwigia glandulosa* Walter subsp. *glandulosa* 2 | USA, Virginia, Norfolk Co. | Tsai-Wen Hsu 11378 | OK338563* | OK381703* |
| *Ludwigia glandulosa* Walter subsp. *glandulosa* 3 | USA, Oklahoma, Coal Co. | Tsai-Wen Hsu 11389 | OK338561* | -- |
| *Ludwigia glandulosa* Walter subsp. *glandulosa* 4 | USA, Alabama, Escombia Co. | Ching-I Peng 19319 | OK338562* | OK381717* |
| *Ludwigia glandulosa* Walter subsp. *glandulosa* 5 | USA, South Carolina, Berkeley Co. | Ching-I Peng 19417 | OK338560* | OK381727* |
| *Ludwigia glandulosa* subsp. *brachycarpa* (Torr. & A. Gray) C.I. Peng 1 | USA, Texas, Red River Co. | Tsai-Wen Hsu 11390 | OK338567* | OK381713* |
| *Ludwigia glandulosa* subsp. *brachycarpa* (Torr. & A. Gray) C.I. Peng 2 | USA, Texas, Red River Co. | Tsai-Wen Hsu 11390 | OK338565* | OK381704* |
| *Ludwigia glandulosa* subsp. *brachycarpa* (Torr. & A. Gray) C.I. Peng 3 | USA, Texas, Red River Co. | Tsai-Wen Hsu 11390 | OK338566* | OK381706* |
| *Ludwigia lanceolata* Elliott 1 | USA, Florida, Walton Co. | Tsai-Wen Hsu 11230 | OK338581* | OK381747* |
| *Ludwigia lanceolata* Elliott 2 | USA, Florida, Clay Co. | Ching-I Peng 19376 | OK338580* | OK381711* |
| *Ludwigia lanceolata* Elliott 3 | USA, Florida, Jefferson Co. | Tsai-Wen Hsu 11265 | OK338619* | -- |
| *Ludwigia lanceolata* Elliott 4 | USA, Florida, Jackson Co. | Tsai-Wen Hsu 11239 | -- | OK381748* |
| *Ludwigia linearis* Walter 1 | USA, Alabama, SR 55 | Tsai-Wen Hsu 11217 | OK338597* | OK381695* |
| *Ludwigia linearis* Walter 2 | USA, Florida, Walton Co. | Tsai-Wen Hsu 11228 | OK338599* | OK381696* |
| *Ludwigia linearis* Walter 3 | USA, Florida, Levy Co. | Tsai-Wen Hsu 11276 | OK338598* | -- |
| *Ludwigia linearis* Walter 4 | USA, Alabama, Escambia Co. | Ching-I Peng 19320 | OK338594* | OK381752* |
| *Ludwigia linearis* Walter 5 |  |  | -- | FN263211 |
| *Ludwigia linifolia* Poir. 1 | USA, Florida, Walton Co. | Tsai-Wen Hsu 11229 | OK338592* | -- |
| *Ludwigia linifolia* Poir. 2 | USA, Florida, Frankalin Co. | Tsai-Wen Hsu 11259 | OK338593* | OK381751* |
| *Ludwigia linifolia* Poir. 3 | USA, Florida, Frankalin Co. | Tsai-Wen Hsu 11259 | OK338591* | OK381750* |
| *Ludwigia linifolia* Poir. 4 | USA, Florida, Putnam Co. | Ching-I Peng 19368 | OK338596* | -- |
| *Ludwigia linifolia* Poir. 5 | USA, Florida, Clay Co. | Ching-I Peng 19371 | OK338590* | OK381749* |
| *Ludwigia linifolia* Poir. 6 |  |  | -- | FN263213 |
| *Ludwigia microcarpa* Michx. 1 | USA, Florida, Gulf Co. | Tsai-Wen Hsu 11254 | OK338603* | OK381740* |
| *Ludwigia microcarpa* Michx. 2 | USA, Florida, Levy Co. | Tsai-Wen Hsu 11278 | OK338602* | OK381742* |
| *Ludwigia microcarpa* Michx. 3 | USA, Florida, Citrus Co. | Tsai-Wen Hsu 11280 | OK338600* | OK381739* |
| *Ludwigia microcarpa* Michx. 4 | USA, Florida, Hernando Co. | Tsai-Wen Hsu 11284 | OK338605* | -- |
| *Ludwigia microcarpa* Michx. 5 | USA, Florida, Hillsboroyb Co. | Tsai-Wen Hsu 11288 | OK338604* | OK381743* |
| *Ludwigia microcarpa* Michx. 6 | USA, Florida, Sarasota Co. | Tsai-Wen Hsu 11294 | OK338601* | OK381741* |
| *Ludwigia microcarpa* Michx. 7 |  |  | -- | FN263212 |
| *Ludwigia ovalis* Miq. 1 |  |  | AB985733 | FN263204 |
| *Ludwigia ovalis* Miq. 2 |  |  | FN263219 | FN263205 |
| *Ludwigia ovalis* Miq. 3 |  |  | FN263220 | FN263206 |
| *Ludwigia ovalis* Miq. 4 |  |  | FN263221 | FN263207 |
| *Ludwigia ovalis* Miq. 5 |  |  | KX168359 | FN263208 |
| *Ludwigia ovalis* Miq. 6 |  |  | -- | FN263209 |
| *Ludwigia ovalis* Miq. 7 |  |  | -- | FN263210 |
| *Ludwigia palustris* (L.) Elliott 1 |  |  | FN263230 | FN263192 |
| *Ludwigia palustris* (L.) Elliott 2 |  |  | FN263231 | FN263193 |
| *Ludwigia palustris* (L.) Elliott 3 |  |  | FN263233 | FN263194 |
| *Ludwigia palustris* (L.) Elliott 4 |  |  | FN263234 | -- |
| *Ludwigia palustris* (L.) Elliott 5 |  |  | KX168360 | -- |
| *Ludwigia palustris* (L.) Elliott 6 |  |  | KX168361 | -- |
| *Ludwigia pilosa* Walter 1 | USA, Alabama, SR 55 | Tsai-Wen Hsu 11216 | -- | OK381728* |
| *Ludwigia pilosa* Walter 2 | USA, Florida, Walton Co. | Tsai-Wen Hsu 11231 | OK338586* | OK381729* |
| *Ludwigia pilosa* Walter 3 | USA, North Carolina, Bladen Co. | Tsai-Wen Hsu 11366 | OK338587* | OK381730* |
| *Ludwigia pilosa* Walter 4 | USA, Alabama, Escambia Co. | Ching-I Peng 19318 | OK338621* | -- |
| *Ludwigia pilosa* Walter 5 | USA, North Carolina, Robeson Co. | Ching-I Peng 19341 | OK338585* | OK381735* |
| *Ludwigia pilosa* Walter 6 | USA, Florida, Putnam Co. | Tsai-Wen Hsu 11358 | OK338582* | OK381726* |
| *Ludwigia pilosa* Walter 7 | USA, Florida, Putnam Co. | Tsai-Wen Hsu 11358 | OK338583* | -- |
| *Ludwigia pilosa* Walter 8 | USA, Florida, Putnam Co. | Tsai-Wen Hsu 11358 | OK338584* | -- |
| *Ludwigia polycarpa* Short & R.Peter 1 | USA, Missouri, Frankin Co. | Tsai-Wen Hsu 11373 | OK338624* | OK381746* |
| *Ludwigia polycarpa* Short & R.Peter 2 | USA, Massachusetts, Hampshire Co. | Tsai-Wen Hsu 11382 | OK338622* | OK381745* |
| *Ludwigia polycarpa* Short & R.Peter 3 | USA, Indiana, Jackson Co. | Tsai-Wen Hsu 11385 | OK338625* | OK381709* |
| *Ludwigia polycarpa* Short & R.Peter 4 | USA, Indiana, Laurence Co. | Tsai-Wen Hsu 11386 | OK338623* | OK381733* |
| *Ludwigia ravenii* C.I Peng 1 | USA, Virginia, Chesapeake | Tsai-Wen Hsu 11379 | OK338588* | OK381707* |
| *Ludwigia ravenii* C.I Peng 2 | USA, Virginia, Chesapeake | Tsai-Wen Hsu 11379 | OK338589* | OK381708* |
| *Ludwigia ravenii* C.I Peng 3 | USA, Virginia, Chesapeake | Tsai-Wen Hsu 11379 | -- | OK381724* |
| *Ludwigia ravenii* C.I Peng 4 | USA, South Carolina, Berkeley Co. | Ching-I Peng 19415 | -- | OK381725* |
| *Ludwigia repens* J.R. Forst. 1 |  |  | FN263222 | FN263198 |
| *Ludwigia repens* J.R. Forst. 2 |  |  | FN263224 | FN263199 |
| *Ludwigia repens* J.R. Forst. 3 |  |  | FN263225 | FN263200 |
| *Ludwigia repens* J.R. Forst. 4 |  |  | FN263228 | -- |
| *Ludwigia repens* J.R. Forst. 5 |  |  | FN263229 | -- |
| *Ludwigia repens* J.R. Forst. 6 |  |  | FN263240 | -- |
| *Ludwigia repens* J.R. Forst. 7 |  |  | FN263241 | -- |
| *Ludwigia repens* J.R. Forst. 8 |  |  | KX168373 | -- |
| *Ludwigia repens* J.R. Forst. 9 |  |  | KX168374 | -- |
| *Ludwigia simpsonii* Chapm. 1 | USA, Florida, Sarasota Co. | Tsai-Wen Hsu 11295 | OK338606* | OK381718* |
| *Ludwigia simpsonii* Chapm. 2 | USA, Florida, Lee Co. | Tsai-Wen Hsu 11298 | OK338613* | OK381722* |
| *Ludwigia simpsonii* Chapm. 3 | USA, Florida, Okechobee Co. | Tsai-Wen Hsu 11334 | OK338607* | -- |
| *Ludwigia simpsonii* Chapm. 4 |  |  | -- | FN263214 |
| *Ludwigia spathulata* Torr. & A. Gray 1 |  |  | FN263232 | FN263201 |
| *Ludwigia spathulata* Torr. & A. Gray 2 |  |  | KX168379 | FN263202 |
| *Ludwigia spathulata* Torr. & A. Gray 3 |  |  | -- | FN263203 |
| *Ludwigia sphaerocarpa* Elliott 1 | USA, Florida, Jackson Co. | Tsai-Wen Hsu 11240 | OK338617* | OK381736* |
| *Ludwigia sphaerocarpa* Elliott 2 | USA, Florida, Lafayette Co. | Tsai-Wen Hsu 11270 | OK338616* | OK381738* |
| *Ludwigia sphaerocarpa* Elliott 3 | USA, Florida, Levy Co. | Tsai-Wen Hsu 11277 | OK338615* | OK381737* |
| *Ludwigia sphaerocarpa* Elliott 4 | USA, Florida, Levy Co. | Tsai-Wen Hsu 11277 | OK338614* | OK381712* |
| *Ludwigia sphaerocarpa* Elliott 5 | USA, Florida, Madison Co. | Tsai-Wen Hsu s.n. | OK338618* | OK381710* |
| *Ludwigia stricta* (C. Wright ex Griseb.) C. Wright 1 | Cuba, Caribbean | K. Rostański 2028 | -- | -- |
| *Ludwigia stricta* (C. Wright ex Griseb.) C. Wright 2 | Cuba, Caribbean | K. Rostański 2028 | -- | -- |
| *Ludwigia stricta* (C. Wright ex Griseb.) C. Wright 3 | Cuba, Caribbean | K. Rostański 2028 | -- | -- |
| *Ludwigia suffruticosa* Walter 1 | USA, Florida, Jackson Co. | Tsai-Wen Hsu 11237 | OK338577* | OK381744* |
| *Ludwigia suffruticosa* Walter 2 | USA, Florida, Lafayette Co. | Tsai-Wen Hsu 11268 | OK338576* | OK381731* |
| *Ludwigia suffruticosa* Walter 3 | USA, Florida, Levy Co. | Tsai-Wen Hsu 11275 | OK338579* | OK381734* |
| *Ludwigia suffruticosa* Walter 4 | USA, Florida, Putnam Co. | Tsai-Wen Hsu 11357 | OK338578* | OK381732* |
| *Ludwigia suffruticosa* Walter 5 | USA, Florida, Jackson Co. | Ching-I Peng 19337 | OK338620* | -- |
