# Supplementary material for: New insights into polyploid evolution and dynamic nature of Ludwigia section Isnardia (Onagraceae)
Source: Bot Stud. 2023 Jun 3;64:14. doi: 10.1186/s40529-023-00387-8 (PMC10239408; doi:10.1186/s40529-023-00387-8)
Supplement: Supplementary file 5 — Additional file 5. One-way ANOVA analyses and student’s t-tests show that the genetic diversities of Isnardia taxa had no significant differences among ploidy levels and sample sizes. [file 40529_2023_387_MOESM5_ESM.docx]

**Additional File 5.** One-way ANOVA analyses and student’s t-tests show that the genetic diversities of *Isnardia* taxa have no significant difference (a) among ploidy levels in ITS region, (b) among sample sizes of each taxon in ITS region, (c) among ploidy levels in *atp*B-*rbc*L region, and (d) among sample sizes of each taxon in *atp*B-*rbc*L region.

(a)

ANOVA

|  | Sum of Squares | df | Mean Square | F | P-value |
| --- | --- | --- | --- | --- | --- |
| Between Groups | .000127 | 4 | .000032 | 1.178971 | .339289 |
| Within Groups | .000836 | 31 | .000027 |  |  |
| Total | .000963 | 35 |  |  |  |

Student’s t-tests

|  | p-value |
| --- | --- |
| 2x vs. polyploid | 0.8095 |
| 2x vs. 4x | 0.9160 |
| 2x vs. 6x | 0.5170 |
| 4x vs. 6x | 0.5063 |

(b)

ANOVA

|  | Sum of Squares | df | Mean Square | F | P-value |
| --- | --- | --- | --- | --- | --- |
| Between Groups | 0.000129505 | 6 | 2.15841E-05 | 0.661359436 | 0.682060325 |
| Within Groups | 0.000424268 | 13 | 3.2636E-05 |  |  |
| Total | 0.000553772 | 19 |  |  |  |

(c)

ANOVA

|  | Sum of Squares | df | Mean Square | F | P-value |
| --- | --- | --- | --- | --- | --- |
| Between Groups | .000050 | 4 | .000012 | .646452 | .633580 |
| Within Groups | .000597 | 31 | .000019 |  |  |
| Total | .000646 | 35 |  |  |  |

Student’s t-tests

|  | p-value |
| --- | --- |
| 2x vs. polyploid | 0.0778 |
| 2x vs. 4x | 0.0744 |
| 2x vs. 6x | 0.6016 |
| 4x vs. 6x | 0.1132 |

(d)

ANOVA

|  | Sum of Squares | df | Mean Square | F | P-value |
| --- | --- | --- | --- | --- | --- |
| Between Groups | 4.48688E-05 | 6 | 7.47814E-06 | 0.312436689 | 0.918338153 |
| Within Groups | 0.000287219 | 12 | 2.39349E-05 |  |  |
| Total | 0.000332088 | 18 |  |  |  |
