# Supplementary material for: New insights into polyploid evolution and dynamic nature of Ludwigia section Isnardia (Onagraceae)
Source: Bot Stud. 2023 Jun 3;64:14. doi: 10.1186/s40529-023-00387-8 (PMC10239408; doi:10.1186/s40529-023-00387-8)
Supplement: Supplementary file 7 — Additional file 7. The estimated migration rates of every two taxa in Ludwigia sect. Isnardia. [file 40529_2023_387_MOESM7_ESM.docx]

**Additional File 7.** The estimated migration rates of every two taxa in *Ludwigia* sect. *Isnardia* based on the nuclear ITS region (a) and chloroplast *atp*B-*rbc*L region (b). Numbers in the matrixes to the power of nine are the migration rates per gene copy per generation (M) for every two *Isnardia* taxa. Matrix shows migration rates from taxon 0 to taxon 1. Taxa are abbreviated as the first three or four letters of species names, e.g., *Ludwigia alata* shows as ala.

(a)

|  | | **Taxon 0** | | | | | | | | | | | | | | | | | | | |
| --- | --- | --- | --- | --- | --- | --- | --- | --- | --- | --- | --- | --- | --- | --- | --- | --- | --- | --- | --- | --- | --- |
|  |  | **ala** | **arc** | **bre** | **cur** | **glab** | **glag** | **lan** | **line** | **lini** | **mic** | **ova** | **pal** | **pil** | **pol** | **rav** | **rep** | **sim** | **spa** | **sph** | **suf** |
| **Taxon 1** | **ala** |  | 1.88 | 1.32 | 1.92 | 2.00 | 1.63 | 1.94 | 1.69 | 1.84 | 2.06 | 1.90 | 1.55 | 1.49 | 1.74 | 1.98 | 0.94 | 1.96 | 2.23 | 1.35 | 1.72 |
|  | **arc** | 1.36 |  | 2.64 | 2.07 | 2.10 | 1.78 | 2.19 | 2.11 | 2.10 | 2.22 | 2.03 | 1.69 | 1.94 | 2.01 | 2.17 | 2.73 | 2.20 | 2.29 | 1.74 | 2.01 |
|  | **bre** | 1.25 | 2.69 |  | 2.00 | 2.07 | 1.71 | 2.11 | 1.95 | 1.97 | 2.14 | 1.97 | 1.63 | 1.83 | 1.91 | 2.13 | 2.12 | 2.11 | 2.29 | 1.53 | 1.88 |
|  | **cur** | 1.20 | 1.82 | 1.41 |  | 2.03 | 1.71 | 1.99 | 1.65 | 1.76 | 1.99 | 1.85 | 1.75 | 1.78 | 1.73 | 1.93 | 1.10 | 3.13 | 2.19 | 1.65 | 1.77 |
|  | **glab** | 1.42 | 2.13 | 1.60 | 2.08 |  | 2.00 | 2.39 | 2.10 | 2.10 | 2.15 | 2.00 | 1.78 | 2.13 | 2.04 | 2.16 | 1.46 | 2.28 | 2.31 | 1.91 | 2.16 |
|  | **glag** | 1.30 | 2.03 | 1.48 | 2.10 | 2.26 |  | 2.45 | 1.98 | 2.02 | 2.14 | 2.01 | 1.77 | 2.13 | 2.00 | 2.14 | 1.21 | 2.36 | 2.35 | 1.84 | 2.16 |
|  | **lan** | 1.44 | 2.25 | 1.61 | 2.18 | 2.22 | 1.88 |  | 2.25 | 2.11 | 2.25 | 2.15 | 1.85 | 2.32 | 2.04 | 2.22 | 1.49 | 2.38 | 2.41 | 2.41 | 2.53 |
|  | **line** | 1.33 | 2.18 | 1.53 | 2.08 | 2.13 | 1.74 | 2.27 |  | 2.65 | 2.22 | 2.05 | 1.68 | 1.92 | 2.01 | 2.21 | 1.28 | 2.30 | 2.35 | 1.73 | 2.09 |
|  | **lini** | 1.35 | 2.05 | 1.51 | 2.03 | 2.07 | 1.69 | 2.21 | 2.61 |  | 2.16 | 1.98 | 1.66 | 1.85 | 1.92 | 2.11 | 1.25 | 2.12 | 2.25 | 1.63 | 1.95 |
|  | **mic** | 1.15 | 1.70 | 1.36 | 1.78 | 1.86 | 1.53 | 1.73 | 1.43 | 1.63 |  | 1.77 | 1.48 | 1.51 | 1.73 | 1.85 | 1.09 | 1.82 | 2.05 | 1.39 | 1.62 |
|  | **ova** | 1.23 | 1.85 | 1.41 | 1.87 | 1.97 | 1.64 | 1.94 | 1.71 | 1.82 | 2.01 |  | 1.63 | 1.66 | 1.76 | 1.94 | 1.14 | 1.92 | 2.17 | 1.48 | 1.75 |
|  | **pal** | 1.25 | 1.97 | 1.45 | 2.09 | 2.13 | 1.79 | 2.35 | 1.82 | 1.91 | 2.08 | 2.00 |  | 2.00 | 2.08 | 2.04 | 1.13 | 2.20 | 2.51 | 1.78 | 2.10 |
|  | **pil** | 1.19 | 1.92 | 1.42 | 2.04 | 2.05 | 1.70 | 2.31 | 1.76 | 1.86 | 2.12 | 1.96 | 1.67 |  | 1.76 | 1.98 | 1.06 | 2.15 | 2.22 | 1.65 | 2.14 |
|  | **pol** | 1.36 | 2.15 | 1.56 | 2.10 | 2.15 | 1.79 | 2.24 | 2.11 | 2.10 | 2.20 | 2.02 | 1.82 | 1.99 |  | 2.18 | 1.35 | 2.24 | 2.37 | 1.71 | 2.02 |
|  | **rav** | 1.53 | 2.36 | 1.68 | 2.15 | 2.21 | 1.86 | 2.41 | 2.37 | 2.30 | 2.28 | 2.11 | 1.79 | 2.14 | 2.20 |  | 1.62 | 2.40 | 2.39 | 1.96 | 2.22 |
|  | **rep** | 1.13 | 2.89 | 1.69 | 1.99 | 2.07 | 1.66 | 2.06 | 1.78 | 1.86 | 2.13 | 1.96 | 1.60 | 1.62 | 1.80 | 2.05 |  | 2.09 | 2.31 | 1.38 | 1.83 |
|  | **sim** | 1.45 | 2.22 | 1.64 | 2.46 | 2.16 | 1.83 | 2.37 | 2.26 | 2.17 | 2.27 | 2.09 | 1.79 | 2.22 | 2.10 | 2.25 | 1.47 |  | 2.37 | 1.96 | 2.17 |
|  | **spa** | 1.55 | 2.24 | 1.70 | 2.09 | 2.14 | 1.87 | 2.39 | 2.25 | 2.20 | 2.18 | 2.04 | 2.21 | 2.19 | 2.22 | 2.23 | 1.69 | 2.32 |  | 2.02 | 2.20 |
|  | **sph** | 1.26 | 2.14 | 1.47 | 2.09 | 2.18 | 1.77 | 2.72 | 2.06 | 2.04 | 2.22 | 2.10 | 1.73 | 2.18 | 1.95 | 2.17 | 1.17 | 2.34 | 2.42 |  | 2.46 |
|  | **suf** | 1.31 | 2.10 | 1.53 | 2.12 | 2.15 | 1.79 | 2.48 | 2.02 | 2.04 | 2.22 | 2.08 | 1.74 | 2.11 | 1.92 | 2.13 | 1.28 | 2.20 | 2.34 | 2.10 |  |

(b)

|  | | **Taxon 0** | | | | | | | | | | | | | | | | | | | |
| --- | --- | --- | --- | --- | --- | --- | --- | --- | --- | --- | --- | --- | --- | --- | --- | --- | --- | --- | --- | --- | --- |
|  |  | **ala** | **arc** | **bre** | **cur** | **glab** | **glag** | **lan** | **line** | **lini** | **mic** | **ova** | **pal** | **pil** | **pol** | **rav** | **rep** | **sim** | **spa** | **sph** | **suf** |
| **Taxon 1** | **ala** |  | 0.43 | 0.48 | 0.49 | 0.43 | 0.46 | 0.43 | 0.47 | 0.47 | 0.46 | 0.24 | 0.37 | 0.57 | 0.36 | 0.48 | 0.41 | 0.51 | 0.46 | 0.40 | 0.37 |
|  | **arc** | 0.47 |  | 0.48 | 0.42 | 0.41 | 0.43 | 0.46 | 0.44 | 0.44 | 0.44 | 0.27 | 0.39 | 0.61 | 0.37 | 0.46 | 0.53 | 0.46 | 0.46 | 0.38 | 0.37 |
|  | **bre** | 0.46 | 0.42 |  | 0.41 | 0.45 | 0.45 | 0.55 | 0.45 | 0.45 | 0.45 | 0.35 | 0.54 | 0.64 | 0.56 | 0.47 | 0.45 | 0.44 | 0.48 | 0.42 | 0.44 |
|  | **cur** | 0.59 | 0.43 | 0.48 |  | 0.46 | 0.47 | 0.54 | 0.47 | 0.47 | 0.47 | 0.28 | 0.38 | 0.60 | 0.40 | 0.49 | 0.42 | 0.52 | 0.45 | 0.43 | 0.45 |
|  | **glab** | 0.49 | 0.41 | 0.49 | 0.44 |  | 0.50 | 0.60 | 0.48 | 0.48 | 0.48 | 0.33 | 0.40 | 0.65 | 0.45 | 0.50 | 0.40 | 0.47 | 0.47 | 0.51 | 0.56 |
|  | **glag** | 0.51 | 0.41 | 0.49 | 0.46 | 0.55 |  | 0.59 | 0.49 | 0.49 | 0.48 | 0.34 | 0.40 | 0.62 | 0.46 | 0.52 | 0.40 | 0.48 | 0.48 | 0.56 | 0.54 |
|  | **lan** | 0.38 | 0.41 | 0.48 | 0.41 | 0.42 | 0.47 |  | 0.46 | 0.46 | 0.47 | 0.26 | 0.39 | 0.61 | 0.38 | 0.46 | 0.39 | 0.45 | 0.45 | 0.44 | 0.41 |
|  | **line** | 0.47 | 0.39 | 0.47 | 0.44 | 0.49 | 0.49 | 0.57 |  | 0.52 | 0.47 | 0.29 | 0.38 | 0.61 | 0.42 | 0.50 | 0.38 | 0.46 | 0.46 | 0.47 | 0.48 |
|  | **lini** | 0.47 | 0.39 | 0.47 | 0.43 | 0.49 | 0.49 | 0.57 | 0.56 |  | 0.47 | 0.26 | 0.38 | 0.61 | 0.42 | 0.50 | 0.38 | 0.46 | 0.46 | 0.47 | 0.48 |
|  | **mic** | 0.46 | 0.39 | 0.47 | 0.43 | 0.49 | 0.48 | 0.53 | 0.47 | 0.47 |  | 0.26 | 0.37 | 0.57 | 0.41 | 0.50 | 0.37 | 0.46 | 0.46 | 0.48 | 0.47 |
|  | **ova** | 0.31 | 0.38 | 0.47 | 0.37 | 0.39 | 0.42 | 0.35 | 0.41 | 0.42 | 0.42 |  | 0.36 | 0.56 | 0.34 | 0.45 | 0.36 | 0.42 | 0.42 | 0.37 | 0.33 |
|  | **pal** | 0.37 | 0.40 | 0.53 | 0.38 | 0.40 | 0.43 | 0.45 | 0.44 | 0.44 | 0.43 | 0.26 |  | 0.60 | 0.51 | 0.45 | 0.40 | 0.43 | 0.45 | 0.38 | 0.37 |
|  | **pil** | 0.31 | 0.38 | 0.44 | 0.36 | 0.38 | 0.38 | 0.33 | 0.39 | 0.40 | 0.39 | 0.26 | 0.37 |  | 0.36 | 0.40 | 0.37 | 0.39 | 0.40 | 0.34 | 0.46 |
|  | **pol** | 0.36 | 0.38 | 0.52 | 0.39 | 0.41 | 0.44 | 0.44 | 0.46 | 0.46 | 0.45 | 0.25 | 0.44 | 0.59 |  | 0.47 | 0.38 | 0.44 | 0.44 | 0.39 | 0.36 |
|  | **rav** | 0.52 | 0.41 | 0.49 | 0.48 | 0.58 | 0.56 | 0.59 | 0.50 | 0.50 | 0.49 | 0.34 | 0.40 | 0.61 | 0.48 |  | 0.40 | 0.49 | 0.50 | 0.57 | 0.55 |
|  | **rep** | 0.45 | 0.51 | 0.49 | 0.42 | 0.40 | 0.43 | 0.45 | 0.44 | 0.44 | 0.43 | 0.26 | 0.40 | 0.60 | 0.38 | 0.45 |  | 0.45 | 0.45 | 0.38 | 0.37 |
|  | **sim** | 0.59 | 0.44 | 0.48 | 0.55 | 0.48 | 0.47 | 0.56 | 0.47 | 0.47 | 0.47 | 0.32 | 0.39 | 0.62 | 0.43 | 0.49 | 0.43 |  | 0.46 | 0.46 | 0.48 |
|  | **spa** | 0.47 | 0.44 | 0.51 | 0.42 | 0.48 | 0.47 | 0.55 | 0.47 | 0.47 | 0.47 | 0.31 | 0.43 | 0.62 | 0.42 | 0.49 | 0.43 | 0.45 |  | 0.45 | 0.47 |
|  | **sph** | 0.48 | 0.41 | 0.50 | 0.44 | 0.54 | 0.56 | 0.60 | 0.49 | 0.49 | 0.49 | 0.31 | 0.39 | 0.61 | 0.44 | 0.51 | 0.39 | 0.47 | 0.47 |  | 0.53 |
|  | **suf** | 0.40 | 0.40 | 0.48 | 0.41 | 0.48 | 0.44 | 0.52 | 0.47 | 0.47 | 0.46 | 0.26 | 0.38 | 0.66 | 0.41 | 0.47 | 0.38 | 0.45 | 0.45 | 0.41 |  |
