# Supplementary material for: New insights into polyploid evolution and dynamic nature of Ludwigia section Isnardia (Onagraceae)
Source: Bot Stud. 2023 Jun 3;64:14. doi: 10.1186/s40529-023-00387-8 (PMC10239408; doi:10.1186/s40529-023-00387-8)
Supplement: Supplementary file 8 — Additional file 8. One-way ANOVA analyses and student’s t-tests for the estimated migration rates between two ploidy levels in Ludwigia sect. Isnardia. [file 40529_2023_387_MOESM8_ESM.docx]

**Additional File 8.** One-way ANOVA analyses and student’s t-tests show that the estimated migration rates between two ploidy levels have (a) significant differences based on ITS region but (b) no significant difference based on *atp*B-*rbc*L region. Groups are coded, showing the direction of the migration rates, e.g., 2×>4× indicates the migration rates from diploids to tetraploids.

(a)

ANOVA

|  | Sum of Squares | df | Mean Square | F | P-value |
| --- | --- | --- | --- | --- | --- |
| Between Groups | 11.853756 | 17 | .697280 | 6.891773 | .000000 |
| Within Groups | 73.858237 | 730 | .101176 |  |  |
| Total | 85.711993 | 747 |  |  |  |

Student’s t-tests

| P-values | 2x>2x | 2x>4x | 2x>6x | 4x>2x | 4x>4x | 4x>6x | 6x>2x | 6x>4x | 6x>6x | 6x>8x | 8x>2x | 8x>4x | 2x>polyploid | polyploid>2x |
| --- | --- | --- | --- | --- | --- | --- | --- | --- | --- | --- | --- | --- | --- | --- |
| 2x>4x | 0.51721 |  |  |  |  |  |  |  |  |  |  |  |  |  |
| 2x>6x | 0.84798 | 0.12418 |  |  |  |  |  |  |  |  |  |  |  |  |
| 2x>8x | 0.27117 | 0.03555 | 0.19463 |  |  |  |  |  |  |  |  |  |  |  |
| 4x>2x | 0.94894 |  |  |  |  |  |  |  |  |  |  |  |  |  |
| 4x>4x | 0.22941 |  |  | 0.00112 |  |  |  |  |  |  |  |  |  |  |
| 4x>6x | 0.74885 |  |  | 0.57803 | 0.03577 |  |  |  |  |  |  |  |  |  |
| 4x>8x | 0.51221 |  |  | 0.13927 | 0.00054 | 0.07036 |  |  |  |  |  |  |  |  |
| 6x>2x | 0.00887 |  |  |  |  |  |  |  |  |  |  |  |  |  |
| 6x>4x | 0.07022 |  |  |  |  |  | 0.15090 |  |  |  |  |  |  |  |
| 6x>6x | 0.04808 |  |  |  |  |  | 0.56058 | 0.53287 |  |  |  |  |  |  |
| 6x>8x | 0.66380 |  |  |  |  |  | 0.70359 | 0.95892 | 0.83182 |  |  |  |  |  |
| 8x>2x | 0.72705 |  |  |  |  |  |  |  |  | 0.59751 |  |  |  |  |
| 8x>4x | 0.27140 |  |  |  |  |  |  |  |  | 0.49319 | 0.31817 |  |  |  |
| 8x>6x | 0.41364 |  |  |  |  |  |  |  |  | 0.49047 | 0.53025 | 0.94836 |  |  |
| 2x>polyploid | 0.74967 |  |  |  |  |  |  |  |  |  |  |  |  |  |
| polyploid>2x | 0.43463 |  |  |  |  |  |  |  |  |  |  |  | 0.00667 |  |
| polyploid>polyploid | 0.91632 |  |  |  |  |  |  |  |  |  |  |  | 0.45959 | 0.02137 |

(b)

ANOVA

|  | Sum of Squares | df | Mean Square | F | P-value |
| --- | --- | --- | --- | --- | --- |
| Between Groups | .041747 | 17 | .002456 | .449504 | .972924 |
| Within Groups | 3.988060 | 730 | .005463 |  |  |
| Total | 4.029807 | 747 |  |  |  |

Student’s t-tests

| P-values | 2x>2x | 2x>4x | 2x>6x | 4x>2x | 4x>4x | 4x>6x | 6x>2x | 6x>4x | 6x>6x | 8x>2x | 8x>4x | 2x>polyploid | polyploid>2x |
| --- | --- | --- | --- | --- | --- | --- | --- | --- | --- | --- | --- | --- | --- |
| 2x>4x | 0.75398 |  |  |  |  |  |  |  |  |  |  |  |  |
| 2x>6x | 0.99484 | 0.63311 |  |  |  |  |  |  |  |  |  |  |  |
| 2x>8x | 0.92511 | 0.91288 | 0.91386 |  |  |  |  |  |  |  |  |  |  |
| 4x>2x | 0.74774 |  |  |  |  |  |  |  |  |  |  |  |  |
| 4x>4x | 0.83470 |  |  | 0.85725 |  |  |  |  |  |  |  |  |  |
| 4x>6x | 0.97799 |  |  | 0.68192 | 0.76819 |  |  |  |  |  |  |  |  |
| 4x>8x | 0.87523 |  |  | 0.93910 | 0.97899 | 0.84989 |  |  |  |  |  |  |  |
| 6x>2x | 0.63053 |  |  |  |  |  |  |  |  |  |  |  |  |
| 6x>4x | 0.70875 |  |  |  |  |  | 0.84726 |  |  |  |  |  |  |
| 6x>6x | 0.31391 |  |  |  |  |  | 0.08937 | 0.08564 |  |  |  |  |  |
| 6x>8x | 0.24749 |  |  |  |  |  | 0.18121 | 0.19599 | 0.46185 |  |  |  |  |
| 8x>2x | 0.16919 |  |  |  |  |  |  |  |  |  |  |  |  |
| 8x>4x | 0.14241 |  |  |  |  |  |  |  |  | 0.99560 |  |  |  |
| 8x>6x | 0.65483 |  |  |  |  |  |  |  |  | 0.24523 | 0.24159 |  |  |
| 2x>polyploid | 0.81967 |  |  |  |  |  |  |  |  |  |  |  |  |
| polyploid>2x | 0.98682 |  |  |  |  |  |  |  |  |  |  | 0.68516 |  |
| polyploid>polyploid | 0.92978 |  |  |  |  |  |  |  |  |  |  | 0.44621 | 0.90831 |
